# Abscisic acid influences ammonium transport via regulation of kinase CIPK23 and ammonium transporters

**DOI:** 10.1093/plphys/kiac315

**Published:** 2022-06-28

**Authors:** Pascal Ganz, Romano Porras-Murillo, Toyosi Ijato, Jochen Menz, Tatsiana Straub, Nils Stührwohldt, Narges Moradtalab, Uwe Ludewig, Benjamin Neuhäuser

**Affiliations:** Institute of Crop Science, Nutritional Crop Physiology, University of Hohenheim, Stuttgart D-70593, Germany; Institute of Crop Science, Nutritional Crop Physiology, University of Hohenheim, Stuttgart D-70593, Germany; Institute of Crop Science, Nutritional Crop Physiology, University of Hohenheim, Stuttgart D-70593, Germany; Institute of Crop Science, Nutritional Crop Physiology, University of Hohenheim, Stuttgart D-70593, Germany; Institute of Crop Science, Nutritional Crop Physiology, University of Hohenheim, Stuttgart D-70593, Germany; Institute of Biology, Plant Physiology and Biochemistry, University of Hohenheim, Stuttgart D-70593, Germany; Institute of Crop Science, Nutritional Crop Physiology, University of Hohenheim, Stuttgart D-70593, Germany; Institute of Crop Science, Nutritional Crop Physiology, University of Hohenheim, Stuttgart D-70593, Germany; Institute of Crop Science, Nutritional Crop Physiology, University of Hohenheim, Stuttgart D-70593, Germany

## Abstract

Ammonium uptake at plant roots is regulated at the transcriptional, posttranscriptional, and posttranslational levels. Phosphorylation by the protein kinase calcineurin B-like protein (CBL)-interacting protein kinase 23 (CIPK23) transiently inactivates ammonium transporters (AMT1s), but the phosphatases activating AMT1s remain unknown. Here, we identified the PP2C phosphatase abscisic acid (ABA) insensitive 1 (ABI1) as an activator of AMT1s in Arabidopsis (*Arabidopsis thaliana*). We showed that high external ammonium concentrations elevate the level of the stress phytohormone ABA, possibly by de-glycosylation. Active ABA was sensed by ABI1-PYR1-like () complexes followed by the inactivation of ABI1, in turn activating CIPK23. Under favorable growth conditions, ABI1 reduced AMT1;1 and AMT1;2 phosphorylation, both by binding and inactivating CIPK23. ABI1 further directly interacted with AMT1;1 and AMT1;2, which would be a prerequisite for dephosphorylation of the transporter by ABI1. Thus, ABI1 is a positive regulator of ammonium uptake, coupling nutrient acquisition to abiotic stress signaling. Elevated ABA reduces ammonium uptake during stress situations, such as ammonium toxicity, whereas ABI1 reactivates AMT1s under favorable growth conditions.

## Teaser

To avoid abiotic stress by ammonium toxicity, Arabidopsis increases ABA levels, starting a complex phospho-regulation of ammonium transport, including the phosphatase ABI1 and the kinase CIPK23.

## Introduction

Uptake of nitrogen from the soil in the form of NO_3_^−^ or ${\text{NH}}_{4}^{+}$ is crucial for plant growth and has to be coordinated with the uptake of other ions, to maintain cellular ion homeostasis and the plasma membrane potential. This complex task is managed on the transcriptional, posttranscriptional, and posttranslational levels by a variety of stimuli and regulators (Krouk et al., 2010). Whereas ammonium uptake in Arabidopsis (*Arabidopsis thaliana*) is subjected to strong transcriptional regulation by light, sugar, and glutamine (Gazzarrini et al., 1999; Rawat et al., 1999), it is additionally regulated and fine-tuned by multiple phosphorylation sites in the ammonium transporter 1 (AMT1) C-termini (Loqué et al., 2007; Neuhäuser et al., 2007; Straub et al., 2017; Wu et al., 2019). Following the identification of a conserved phosphorylation site in AMT1 C-termini (Nuhse et al., 2004), the phosphorylation of the respective T460 (AMT1;1) and T472 (AMT1;2) residues has been shown to inactivate AMT1 transport activity (Loqué et al., 2007; Neuhäuser et al., 2007). This strong inhibition of uptake has been proposed to be an effective mechanism for avoiding ammonium toxicity (Neuhäuser et al., 2007). In agreement with this hypothesis, the phosphorylation of the AMT1 C-termini shows a fast and strong increase after ammonium resupply that is absent in nitrogen-starved plants (Lanquar et al., 2009; Straub et al., 2017). Adjacent to the conserved threonine residue, the AMT1 C-termini contains several additional nonconserved phosphorylation sites that are differentially phosphorylated upon differences in the nitrogen supply (Engelsberger and Schulze, 2012; Menz et al., 2016). In AtAMT1;3, phosphorylation of one of these sites has been shown to fine-tune AtAMT1;3 activity in dependence on nitrate availability (Wu et al., 2019). These nonconserved phosphorylation sites are hypothesized to integrate multiple environmental stimuli with AMT1-mediated ammonium nutrition (Wu et al., 2019).

Whereas the kinases involved in the AMT-specific phosphorylation mechanisms of the nonconserved C-terminal region remain unclear, ammonium-dependent phosphorylation of the conserved C-terminal phosphorylation site has been shown to be mediated by calcineurin B-like protein (CBL)-interacting protein kinase 23 (CIPK23; Straub et al., 2017). Earlier research has highlighted the role of the kinase CIPK23 as an activator of the potassium transporter AKT1 during low K^+^ supply, while it additionally modifies the affinity of the crucial nitrate transporter NPF6;3 (Xu et al., 2006; Ho et al., 2009) and regulates the maintenance of the iron transporter IRT1 at the plasma membrane and its degradation under metal stress (Dubeaux et al., 2018). These processes are carried out via the phosphorylation of the transporters by a CIPK23–CBL complex interacting with the transporters at the plasma membrane (Lee et al., 2007). This suggests that the kinase is crucial for balancing the cation/anion uptake ratio and for the inorganic nitrogen form being taken up. Additionally, the kinase is also involved in the regulation of ammonium uptake by phosphorylation of AtAMT1;1 and AtAMT1;2, resulting in inactive transporters (Straub et al., 2017). Whether the activity of the AMTs is restored after phosphorylation remains unknown.

The Arabidopsis genome contains approximately 150 protein phosphatases, but not all of them are expressed in roots (Kerk et al., 2008). The *ABA-INSENSITIVE1* (*ABI1*) gene encodes a ubiquitously expressed serine/threonine type 2C protein phosphatase that is a member of clade A in the PP2C phylogenetic tree (Xue et al., 2008). This clade consists of nine members. ABI1 and its homolog ABI2 have been identified as key components of abscisic acid (ABA) signaling (Leung et al., 1997). Ammonium toxicity has been genetically linked to ABA signaling (Li et al., 2012). Mutants of both genes are insensitive to ABA, indicating their role as negative regulators in the pathway (Gosti et al., 1999; Merlot et al., 2001).

Members of the PP2C clade A are regulators of calcium-dependent kinases. The binding of the phosphatase to the kinase prevents (auto-)phosphorylation of the kinase. This interaction is canceled when the phosphatase comes into contact with an ABA/ABA–receptor complex formed by PYRABACTIN RESISTANCE 1/PYRABACTIN RESISTANCE 1-Like (PYR/PYL) proteins and ABA. Liberation of the kinase finally leads to its activation and target phosphorylation (Park et al., 2009; Umezawa et al., 2009; Yin et al., 2009). The interaction of ABI1 with SNF1 (Sucrose Non-fermenting 1), SNF1-related protein kinase 2 (OST1), and SnRK2.4 in the absence of ABA is a well-known example leading to kinase inhibition (Yoshida et al., 2006; Lynch et al., 2012; Krzywińska et al., 2016). A homolog to ABI1, ABI2, was even found to be involved in nitrate uptake regulation and interacted with CIPK23, CBL1, and the NPF6.3 nitrate transporter (Leran et al., 2015). Furthermore, HAB1 and HAB2 have been shown to interact with OST1 (Vlad et al., 2009). Interestingly, two clade A PP2Cs, namely PP2CA and AIP1 (At1G07430), are both able to regulate the potassium channel AKT1 by modulating CIPK6 and CIPK16 activity (Lee et al., 2007; Lan et al., 2011). Moreover, AIP1 interacts with AKT1 and is therefore proposed directly to inhibit AKT1 activity by dephosphorylation.

In this study, we have determined that the ABI1 phosphatase regulates ammonium transport and have addressed its role in regulating AMT1 activity in an ammonium- and ABA-dependent manner. We hypothesized that ABI1 stimulates AMT1 activity both by direct dephosphorylation and by CIPK23 inactivation and that ammonium toxicity as an abiotic stress is signaled by increased ABA in the roots. Consequently, the binding of a PYL/ABA complex to ABI1 inhibits AMT1 and CIPK23 dephosphorylation by ABI1, resulting in ammonium-dependent phosphorylation and inactivation of AMT1s by CIPK23.

## Results

### Knockdown mutant *abi1-2* shows reduced methylammonium susceptibility

To identify possible AMT1 activators, we selected phosphatases showing at least moderate expression in roots. With regard to phosphatase families with several candidates, we focused on those that were most strongly expressed (Supplemental Table S1). In an initial screen of 73 Arabidopsis T-DNA insertion lines, we tested these root-expressed phosphatase candidates for reduced sensitivity to toxic ammonium and methylammonium (MeA) conditions. Because of the high variability of the root growth, we initially confirmed that the candidates were also expressed in the hypocotyl. We proceeded with a hypocotyl elongation assay and identified the *abi1-2* knockdown mutant (Wu et al., 2015) and thereby ABI1 (At4G26080) as a potential regulator of AMT1 activity. We first confirmed that the T-DNA insertion in *ABI1* (At4G26080; SALK_072009C) was homozygous and resulted in an ∼90% knockdown of *ABI1* expression (Supplemental Figure S1, a and b). Second, we reproduced the initial screening test (hypocotyl elongation) for Col-0 and *abi1-2* with an increased number of replicates (*n* > 200). This experiment confirmed the reduced ammonium and MeA susceptibility of the *abi1-2* line (Supplemental Figure S2b). T-DNA insertion lines for three other clade A members were also part of our screen (*HAB1*: At1G72770; *HAB2*: At1G17550 and *PP2CA*: At3G11410); however, these did not confer reduced ${\text{NH}}_{4}^{+}$ susceptibility, suggesting specificity of ABI1 in this regulation (Supplemental Figure S2a).

**Figure 1:**
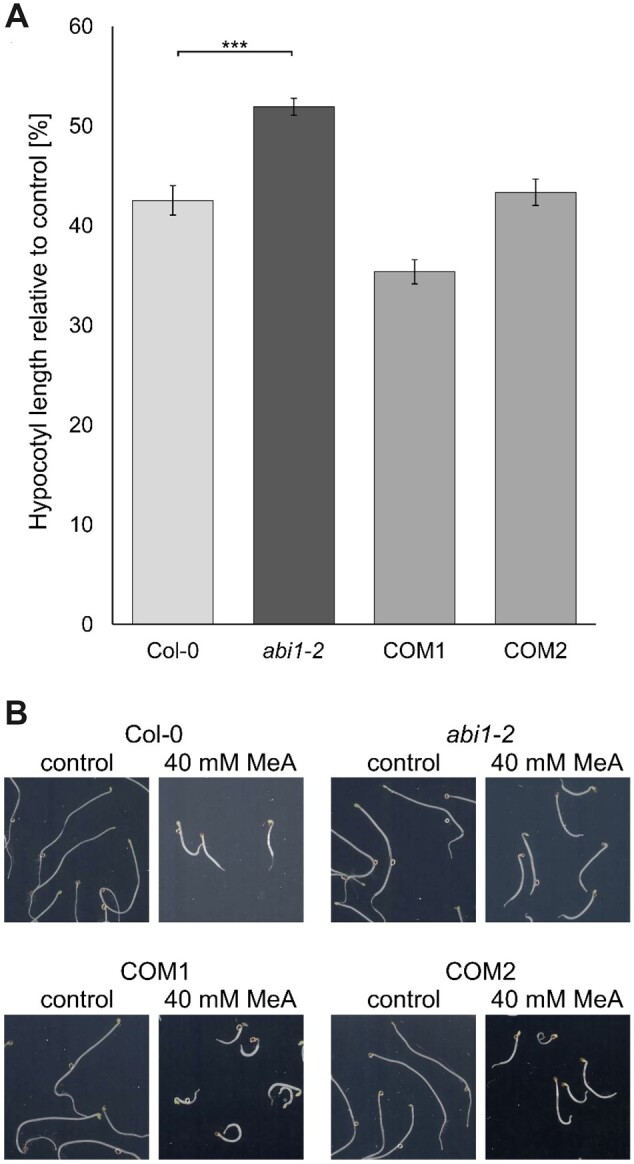
Lower ammonium sensitivity in *abi1-2* plants. A, Hypocotyl length [%] of etiolated Col-0, *abi1-2* and complementation line (COM1 and COM2) seedlings grown on media containing 40-mM MeA relative to growth on control media without MeA. Relative length ±sem of three replicates (*n* ≥ 30). B, Representative images of etiolated seedlings grown under control conditions and in 40-mM MeA. Height and width of the images correspond to 20 mm. Statistical significance was tested by an ANOVA followed by a pairwise comparison, significant differences are indicated by ^***^P < 0.001.

To establish the specificity of the effect in the *abi1-2* line, we created two independent complementation lines; these slightly overexpressed *ABI1* and from here on are referred to as COM1 and COM2. All lines were again tested under toxic MeA conditions in the hypocotyl elongation assay (Figure 1, A and B). The mutant *abi1-2* line again showed longer hypocotyls under toxic MeA conditions compared with those of Col-0 plants, whereas both complementation lines suffered from slightly increased susceptibility (Figure 1, A and B).

### Nitrogen concentration is slightly reduced in the *abi1-2* shoots

**Figure 2:**
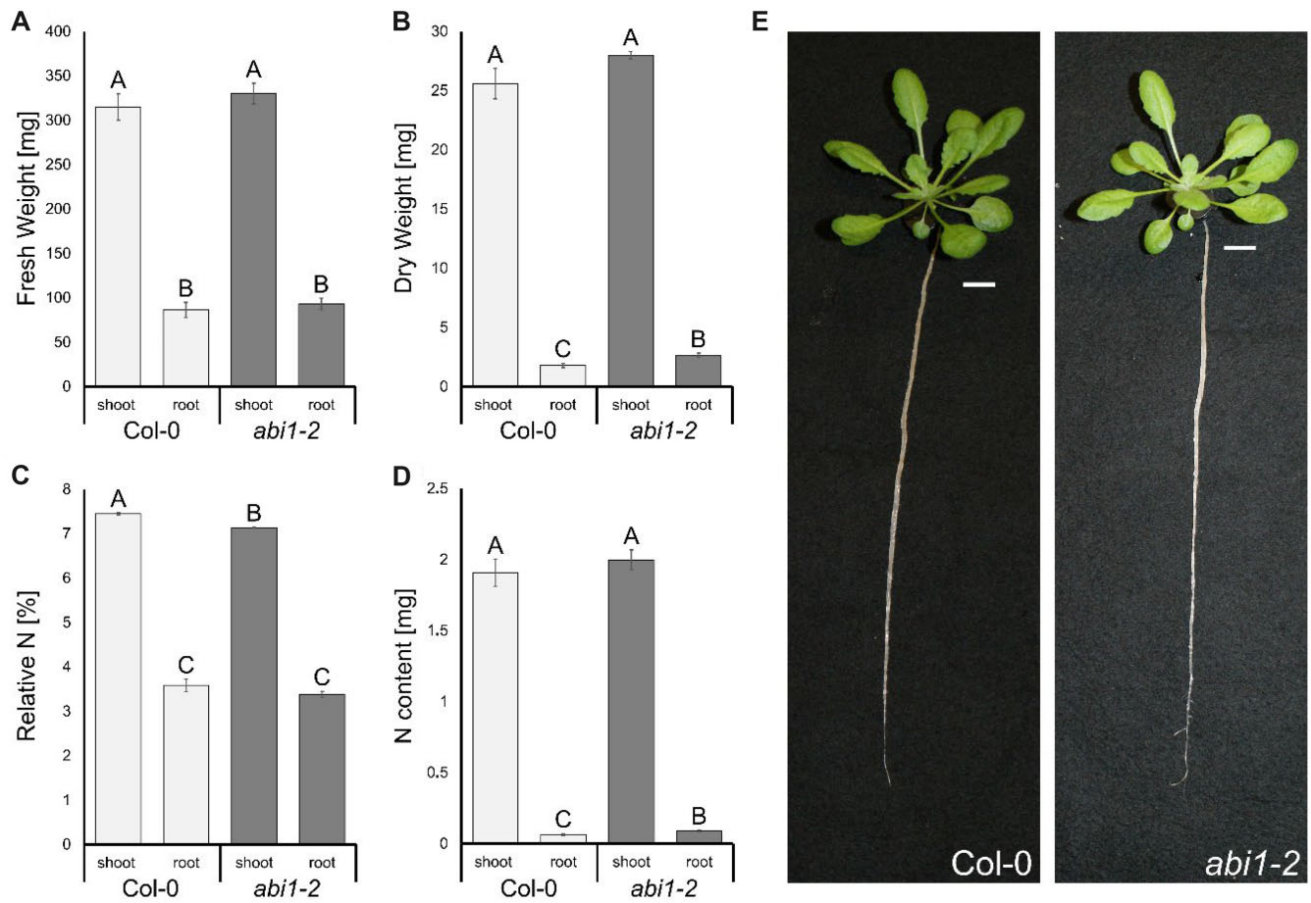
Minor growth effect of the *ABI1* knockout under nonstressed conditions but slightly reduced N-concentrations in the shoots of *abi1-2* plants. A, Fresh weight of roots and shoots of 6-week-old Col-0 and *abi1-2* plants. B, Dry weight of roots and shoots of 6-week-old Col-0 and *abi1-2* plants. C, Relative nitrogen in % (w/w) in shoots and roots of 6-week-old Col-0 and *abi1-2* plants. D, Nitrogen content in the roots and shoots of 6-week-old Col-0 and *abi1-2* plants. E, Images of representative 6-week-old Col-0 and *abi1-2* plants grown under hydroponic culture. Data are shown as means ± sem (Standard Error of the Mean) (*n* = 20). Statistical significance was tested by an ANOVA followed by Tukey’s post-hoc test. Significant differences are indicated by capital letters (*P* < 0.05).

To address the ammonium-sensitive phenotype of the *abi1-2* line further, we grew wild-type (WT) and *abi1-2* plants under standard hydroponic conditions supplemented with 2-mM ammonium nitrate for 6 weeks (Figure 2E). Root and shoot fresh weights were similar for WT and *abi1-2* plants, whereas root dry weight was slightly higher in *abi1-2* plants (Figure 2, A and B). The shoot nitrogen content was similar but the nitrogen percentage in the *abi1-2* shoots was reduced (Figure 2, C and D) even under standard growth conditions.

### Ammonium uptake in the *abi1-2* line is impaired

To detect the reason for the increased tolerance of *abi1-2* to toxic ammonium and MeA, physiological ammonium absorption in plants roots was examined by short-term uptake. Plants were grown hydroponically for 6 weeks and subsequently placed in nitrogen-deficient solution for 4 days. This period of N starvation has previously been shown to increase the amount of AMT transcripts and, at the same time, to decrease the phosphorylation of the transporters (Yuan et al., 2007; Lanquar et al., 2009; Straub et al., 2017). The combination of the two effects results in a high ammonium uptake capacity. Starved plants were placed in 0.5 and 5-mM ^15^${\text{NH}}_{4}^{+}$ uptake solution for 10 min and the uptake was quantified by isotope ratio mass spectrometry. To induce ammonium-dependent phosphorylation of AMT1s by CIPK23, a second timepoint for nitrogen uptake analysis was set at 2.5 h after an ammonium shock (20-mM ${\text{NH}}_{4}^{+}$) for 5 min, followed by N starvation conditions.

**Figure 3:**
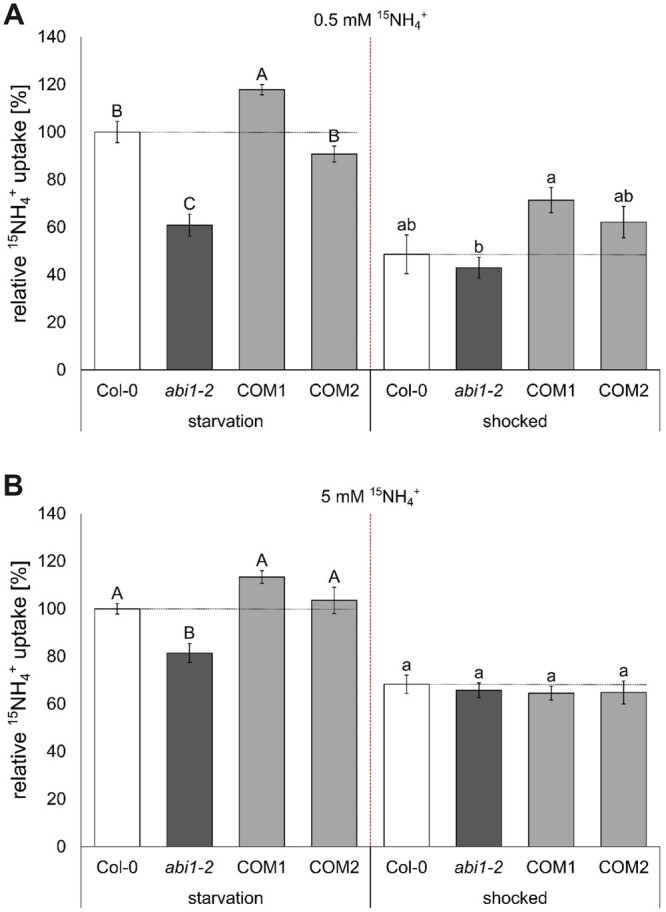
Reduced short-term ammonium uptake in *abi1-2* plants. A, Short-term high affinity (0.5 mM) and (B) low affinity (5 mM) ammonium (^15^${\text{NH}}_{4}^{+}$) uptake (10 min) after N starvation phase (left) and 2.5 h after 5 min (20 mM) ammonium shock (right). Data combine the relative ^15^${\text{NH}}_{4}^{+}$ uptake of three repetitions and was normalized by the mean of Col-0 at starvation of each repetition. Data are shown as mean relative ^15^${\text{NH}}_{4}^{+}$ uptake ±sem (*n* ≥ 9). Statistical significance was tested by an ANOVA followed by Tukey’s post-hoc test. Significant differences are indicated by capital or small letters (*P* < 0.05).

After starvation, most AMTs in the WT and in the complementation lines were expected to be fully de-phosphorylated and active, whereas in the *abi1-2* line, we expected decreased uptake attributable to remaining AMT1 phosphorylation. Accordingly, a lack of dephosphorylation might account for the >40% lower ammonium uptake after starvation in the *abi1-2* line, compared with the WT (Figure 3, A and B). Furthermore, this would imply hyperactivity of the CIPK23 kinase, leading to AMT1 phosphorylation and inactivation.

By subjecting the plants to an ammonium shock, we induced AMT inactivation by CIPK23-dependent phosphorylation, followed by N starvation for reactivation after some time. We then quantified ammonium acquisition after 2.5 additional hours of starvation, following the shock. At this point the *abi1-2* plants showed no difference in ammonium uptake (Figure 3). The COM1 line, however, showed stronger high-affinity ammonium uptake than the mutant line. In agreement with the potential role of ABI1 in the regulation of the high-affinity ammonium transport, the uptake in the low-affinity ammonium range was only reduced by ∼20% compared to the Col-0 plants (Figure 3B), reflecting that uptake at this high concentration is the sum of high and low-affinity ammonium transport and not only AMT1 mediated.

### ABI1 interacts with AMT1;1, AMT1;2, and CIPK23

The reduced ammonium uptake in *abi1-2* after N starvation might be related to higher AMT1 phosphorylation under these conditions. This might be explained by a deregulated and hyperactive CIPK23 kinase or by the inability to dephosphorylate AMT1. In both cases, ABI1 needs to interact with CIPK23 or with the AMT1s. We, therefore, checked whether ABI1 interacted with AMT1s and/or CIPK23.

**Figure 4:**
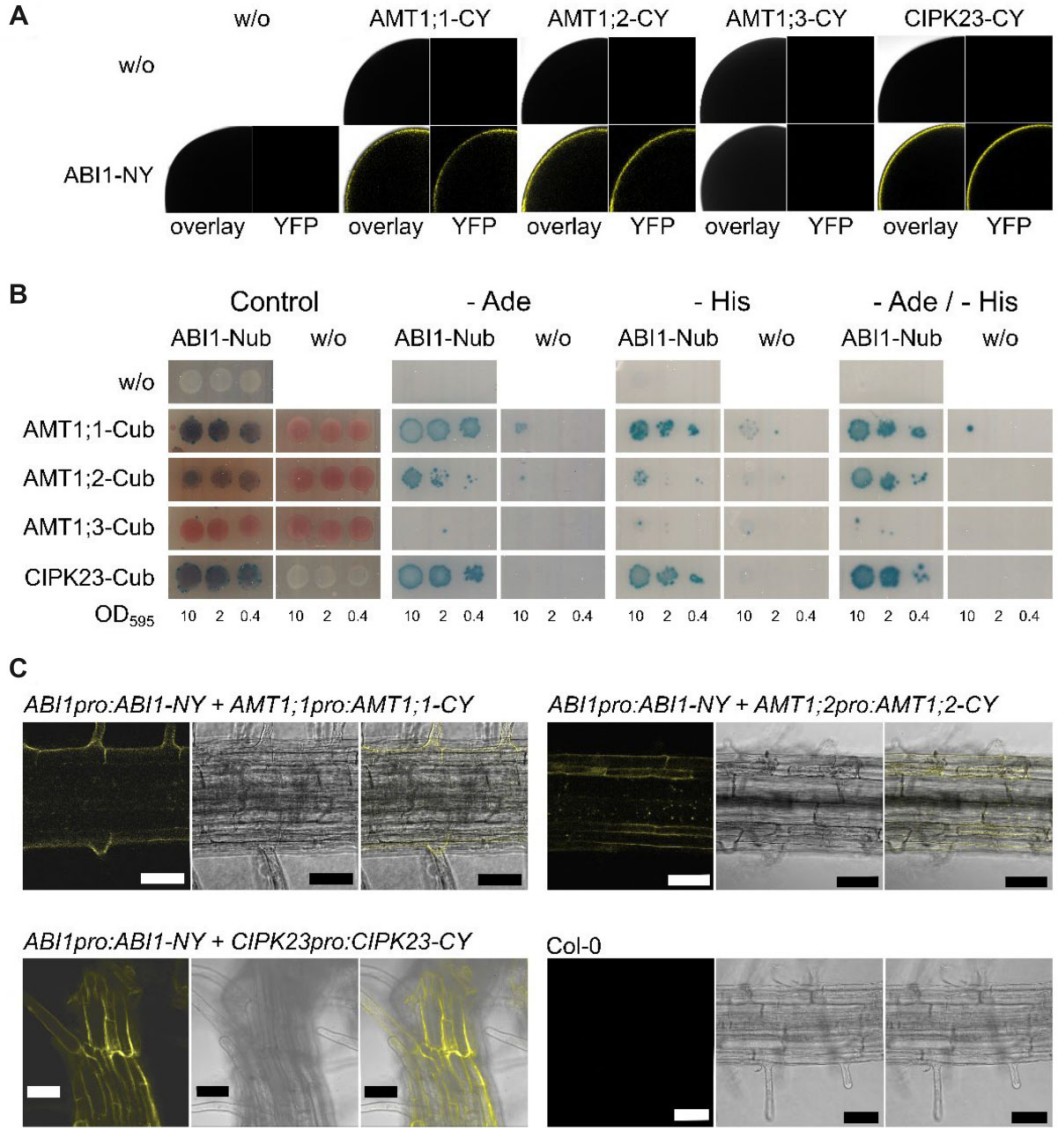
ABI1 interacts with CIPK23, AMT1;1 and AMT1;2. A, Oocytes injected with one or both of the potential interaction partners. Upper row only the potential interaction partner fused to the C-terminal YFP part lower row ABI1 fused to the N-terminal YFP part by itself or together with the putative interaction partner. Left picture = overlay of brightfield image and YFP channel, right picture = only YFP channel. B, Protein interaction of CIPK23 and native AMT1s with ABI1. ABI1 was fused to the N-terminal part of ubiquitin, whereas the interaction partners are fused to the C-terminal part of ubiquitin. Growth on media without adenine and/or histidine and blue staining by X-Gal overlay indicate protein interaction. Figure shows one representative image from four repetitions. C, Representative confocal microscopy pictures of plants stably co-expressing ABI1, AMTs, and CIPK23 fusions carrying split-YFP parts under their endogenous promoter. Upper left, Col-0 control; lower left, ABI1/CIPK23 interaction; upper right, ABI1/AMT1;1 interaction; *lower right*, ABI1/AMT1;2 interaction. From left to right, BiFC-YFP fluorescence signal, brightfield image, overlay. Scale bars indicate 50 µm.

We first tested this interaction using bimolecular fluorescence complementation with a split YFP approach in *Xenopus laevis* oocytes. In this experiment ABI1 showed interaction with CIPK23, AMT1;1, and AMT1;2 but not with AMT1;3 (Figure 4A). This was confirmed by a split ubiquitin assay in yeast. Yeast expressing all protein combinations showed growth on control media, whereas the ABI1/CIPK23, AMT1;1 and AMT1;2, but not AMT1;3, combinations also promoted growth on selective media (Figure 4B). Finally, the physiological importance of the three interactions was supported by an *in planta* bimolecular fluorescence complementation assay (BiFC). BiFC fusion proteins containing either the N- or C-terminal part of YFP were expressed using their endogenous promoters. WT control plants did not exhibit any fluorescence, whereas combinations of *ABI1pro:ABI1-NY* with *CIPK23pro:CIPK23-CY* and with *AMT1;1pro:AMT1;1-CY* and *AMT1;2pro:AMT1;2-CY* all exhibited YFP fluorescence (Figure 4C).

### C-terminal deletions do not inhibit ABI1/AMT interactions

**Figure 5:**
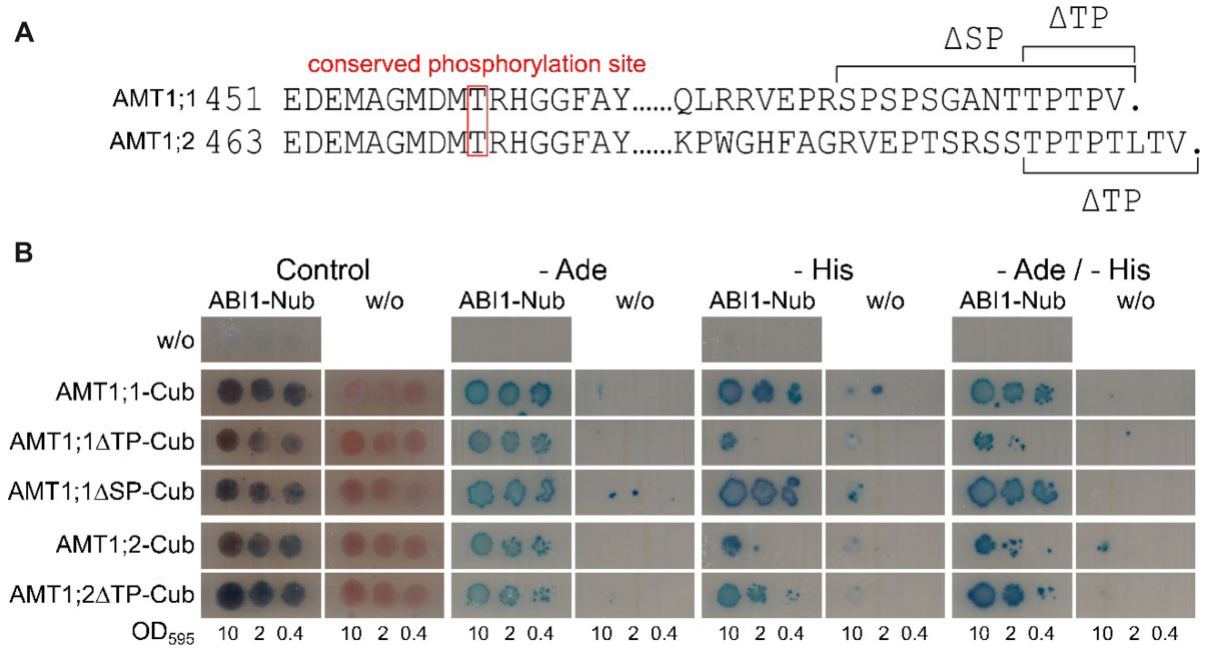
C-terminal deletions do not inhibit ABI1/AMT interactions. A, Sequence alignment of the AMT1;1 and AMT1;2 C-termini. The conserved phosphorylated threonine residue is highlighted by the box. Dots indicate the end of the conserved C-terminal region. Brackets indicate parts removed in the C-terminal truncation mutants. B, Protein interaction of native AMT1s and C-terminal AMT1 truncation mutants with ABI1. ABI1 was fused to the N-terminal part of ubiquitin, whereas the interaction partners are fused to the C-terminal part of ubiquitin. Growth on media without adenine and/or histidine and blue staining by X-Gal overlay indicate protein interaction. Figure shows one representative image from four repetitions.

Several serine and threonine residues in phosphorylation motifs have been found to be differentially phosphorylated in the AMT1 C-termini (Engelsberger and Schulze, 2012; Wu et al., 2019). Although only a single threonine residue is phosphorylated in the conserved part of the C-termini, stretches with phosphorylation motifs occur in the nonconserved AMT1;1 and AMT1;2 protein tails (Figure 5A). The first motif in AMT1;1 (TPTP) starts at position T497, and the second (SPSPS) starts at S488. In AMT1;2, the TPTP motif starts at residue T507. To address which C-terminal segments of the AMTs are of importance for ABI1 binding and might therefore also contain the de-phosphorylation sites targeted by ABI1, we stepwise deleted the respective motifs (Figure 5A).

We then conducted a split-ubiquitin yeast assay to address the effect of the mutations on the ABI1/AMT1 interaction. As indicated above, protein interaction occurred between ABI1 and both native AMTs in the assay (Figure 5B), in agreement with the observations made during the BiFC experiment. Interestingly, the C-terminal deletions in AMT1 did not disrupt protein interactions, since all mutants still interacted similarly with ABI1 (Figure 5B).

### ABI1 specifically affects the phosphorylation state at the conserved AMT1 C-terminus

To assess the phosphorylation status of the AMT1s at both uptake timepoints, we extracted total root protein followed by phosphorylation-sensitive immunodetection of the conserved AMT1 C-terminus. A custom-made phosphorylation-sensitive antibody targeting the threonine in the conserved C-terminal peptide GMDMT(p)RHGGFA of AMT1s was used to check for phosphorylation differences among the lines. Additionally, we used an AMT1;2-specific antibody control and confirmed that AMT1;2 abundance did not differ between the plant lines at each tested timepoint (Supplemental Figure S3).

**Figure 6:**
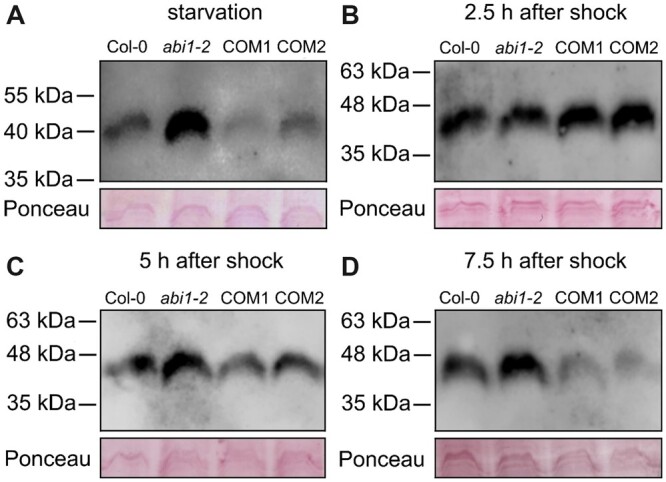
ABI1 affects the phosphorylation status of the conserved AMT1 C-termini. Protein gel blot analysis of total root protein extract from Col-0, *abi1-2* and complementation lines (COM1 and COM2) by using a phosphorylation-specific antibody detecting phosphorylation of the conserved threonine in the AMT1 C-termini after N starvation for 4 days (A), after a nitrogen shock followed by 2.5 h (B), 5 h (C), or 7.5 h (D) of N starvation. Upper part shows detection of phosphorylated AMT1 C-termini and lower part shows loading controls stained with Ponceau red.

After 4 days of N starvation, we were still able to detect phosphorylated AMTs in all lines after 10 min of exposure in an Odyssey Fc chamber (Figure 6A). The strongest phosphorylation was observed in line *abi1-2*, followed by WT Col-0. Both complementation lines showed less AMT1 phosphorylation than Col-0, with line COM1 having the lowest phosphorylation (Supplemental Figure S4a).

The AMT1 phosphorylation of plants that were exposed to 5-min ammonium shock was completely different after 2.5  h. In agreement with previous analyses, the AMT1s were heavily phosphorylated by the shock and this remained in all lines after 2.5 h of additional starvation period (Figure 6B;Supplemental Figure S4B).

At later timepoints, at 5 and 7.5 h after the shock, however, AMT1s were progressively de-phosphorylated, although this depended on the genetic background. At the 5-h timepoint, AMT1 dephosphorylation was slightly more pronounced in the complementation lines than in the WT, whereas AMT1s on the *abi1-2* background were still heavily phosphorylated (Figure 6C;Supplemental Figure S4C). At 7.5 h after the shock, we observed the same phosphorylation pattern as that at the beginning of the experiment (Figure 6D;Supplemental Figure S4D), with AMT1s in *abi1-2* being strongly phosphorylated.

Altered gene expression of the AMT1s or their regulatory genes *CBL1* and *CIPK23* might also account for uptake and phosphorylation differences between the lines. However, real-time quantitative polymerase chain reaction (qPCR) of these genes confirmed that no major changes in gene expression occurred under these conditions. This was confirmed for all relevant genes in all plant lines at all investigated timepoints in several replicate experiments (Supplemental Figure S5). To ensure that AMT1 localization and protein stability were not affected in the *abi1-2* plants, we checked the fluorescence from AMT1;1-GFP and AMT1;2-GFP fusion construct expressing plants under their endogenous AMT1 promoters in the *abi1-2* and Col-0 backgrounds. In both backgrounds, we observed similar AMT-specific plasma membrane localization and GFP signal strength (Supplemental Figure S6).

### Ammonium toxicity increased ABA levels by de-glycosylation

**Figure 7:**
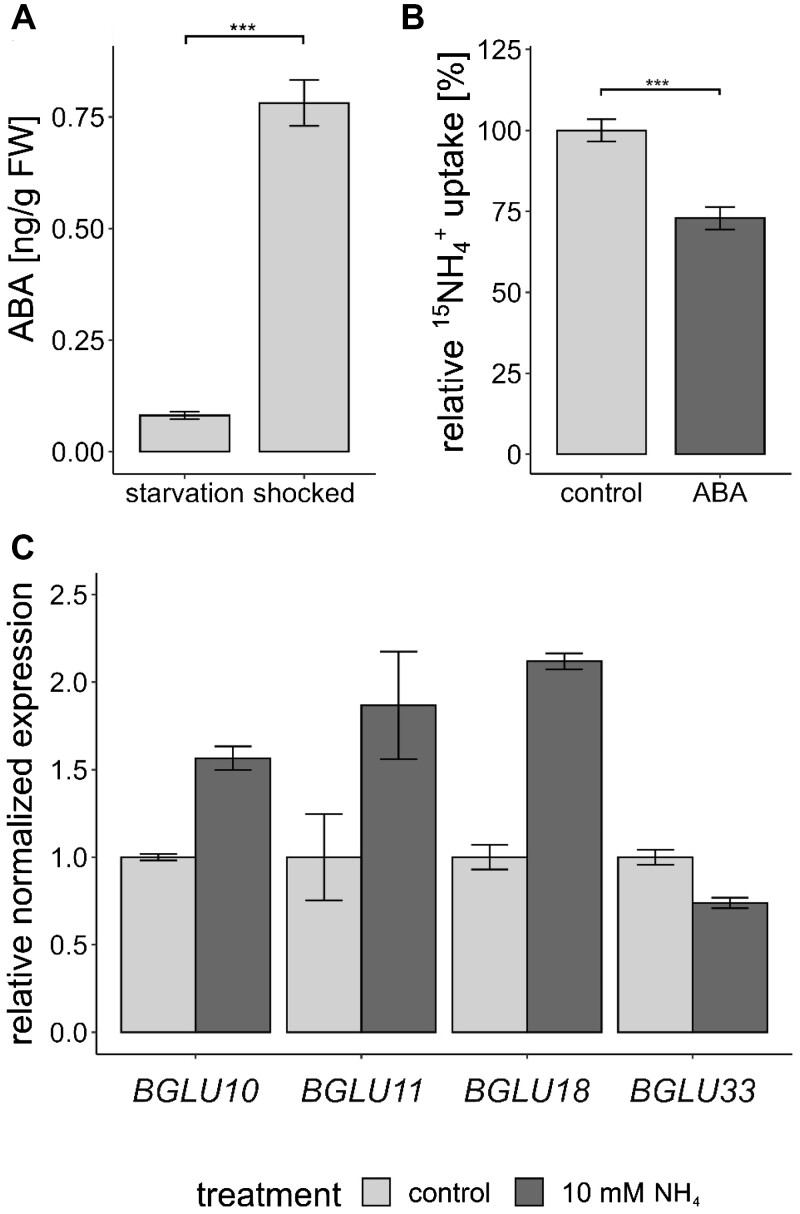
ABA signals are involved in the response to toxic ammonium concentrations. A, ABA concentration ±sem (*n* = 8) in starved and shocked (5 min 20-mM ${\text{NH}}_{4}^{+}$ followed by 2.5 h of starvation) plant roots. Statistical significance was tested by an ANOVA followed by a pairwise comparison, significant differences are indicated by ^***^P < 0.001. B, Short-term ammonium (^15^${\text{NH}}_{4}^{+}$) uptake (10 min, 0.5 mM ^15^${\text{NH}}_{4}^{+}$) in Col-0 plants after preincubation in 100-µM ABA for 1 h. Data combine the relative ^15^${\text{NH}}_{4}^{+}$ uptake of four repetitions and was normalized by the mean of the control of each repetition. Data are shown as mean relative ^15^${\text{NH}}_{4}^{+}$ uptake ±sem (*n* = 19). Statistical significance was tested by an ANOVA followed by a pairwise comparison, significant differences are indicated by ^***^P < 0.001. C, Normalized expression of BGLU genes in starved and shocked (10-mM ${\text{NH}}_{4}^{+}$ for 1 h) plant roots (*n* = 3).

Since ABI1 activity is regulated by ABI1/ABA/PYL complex formation, we further analyzed whether ammonium toxicity, like other abiotic stresses, is also signaled by this complex formation. We tested the interaction of ABI1 and the PYR/PYL ABA-receptor proteins in yeast and identified an interaction between ABI1 and almost all PYL proteins. This interaction tended to increase when we added ABA (Supplemental Figure S7). To closer investigate the involvement of ABA in this regulation, we checked that the ammonium shock performed in the uptake experiment led to a fast and strong increase of ABA in the roots. By comparing the ABA concentration in the roots under N starvation and at the 2.5-h timepoint, a nine-fold increase after the shock was discovered (Figure 7A).

We further tested whether an external ABA supply could directly affect the short-term ammonium uptake of WT plants. Col-0 plants were grown as described above and, after a 4-day starvation period, we exposed the plants to 100-µM ABA for 1 h, after which we quantified the ^15^N ammonium uptake at 0.5 mM for 10 min. Equivalent to the *abi1-2* knockout, the ABA shock strongly reduced the ammonium uptake of the plants (Figure 7B).

Since the root ABA increase appeared relatively rapid, namely only 2.5 h after the ammonium shock, this might be the consequence of ABA activation by de-glycosylation. In order to test this hypothesis, we monitored the expression of the *BGLU* genes involved in ABA de-glycosylation in the endoplasmic reticulum and in the vacuole (Ma et al., 2018). After shocking the plants for 1 h with 10 mM ${\text{NH}}_{4}^{+}$, we observed an increased expression of *BGLU10* and of its homolog *BGLU11.* BGLU10 mediates ABA de-glycosylation in the vacuole. *BLGU33 (BG2)* is also important for vacuolar ABA activation, but did not increase in expression. *BGLU18 (BG1)*, encoding an ER-localized homolog, also showed an ammonium-dependent increase in expression (Figure 7C).

**Figure 8:**
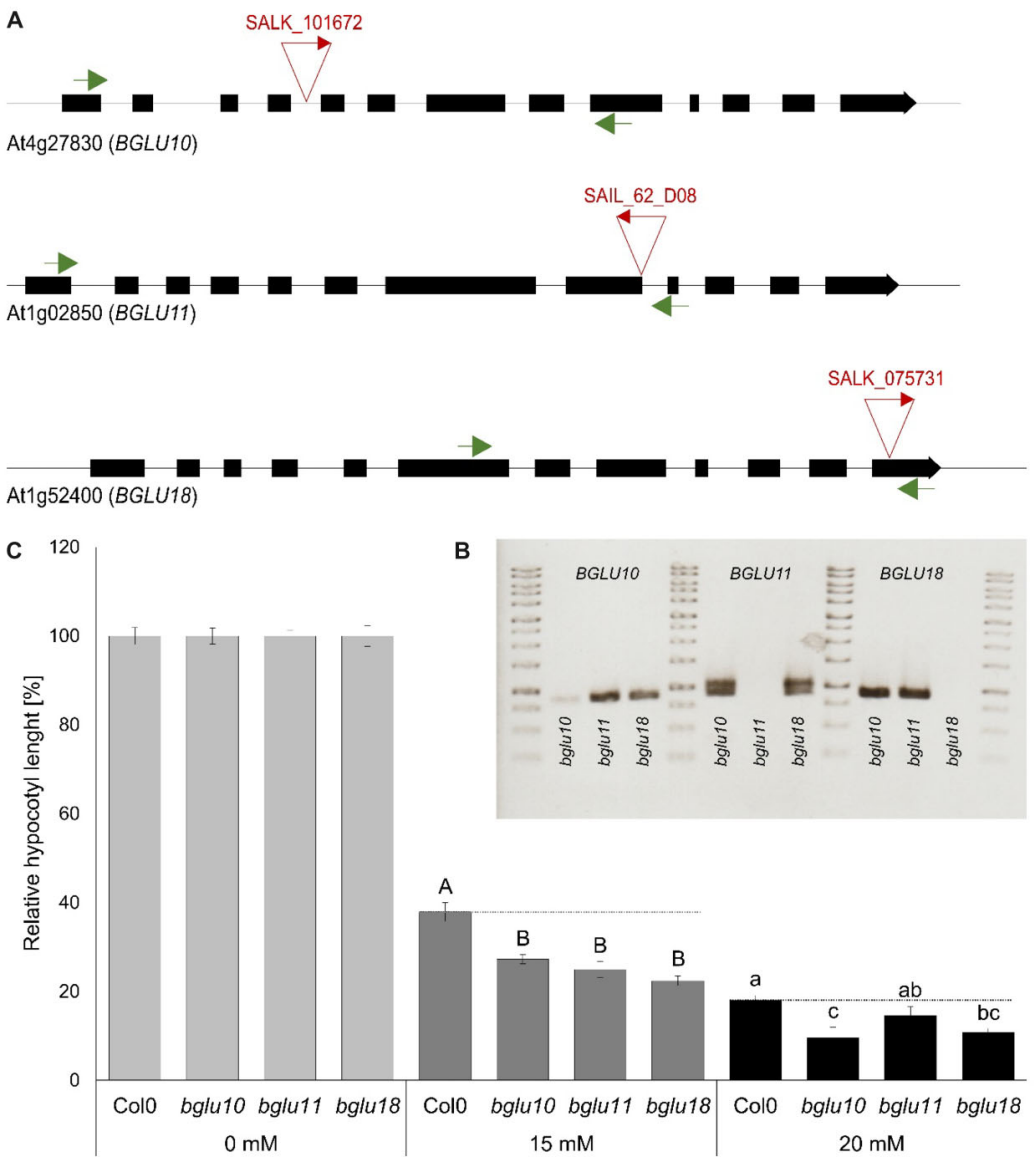
Reduced ammonium toxicity in *bglu* plants. A, Schematic drawing of the *BGUL10* (At4g27830), *BGLU11* (At1g02850), and *BGLU18* (At1g52400) gene structure. Black boxes represent exons, the line represents introns or UTRs (untranslated regions). The place of T-DNA insertion is indicated by triangles. Primers for quantitative PCR spanning the insertion region are indicated by arrows. B, Gel picture of quantitative PCR covering several exons and spanning the T-DNA insertion. C, Representative results of relative hypocotyl length of WT and *bglu* lines in control (0 mM ${\text{NH}}_{4}^{+}$) and toxic (15 and 20-mM ${\text{NH}}_{4}^{+}$) conditions. Data are shown as mean ±sem (*n* = 50). Statistical significance was tested by an ANOVA followed by Tukey’s post-hoc test. Significant differences are indicated by capital or small letters (*P* < 0.05).

We, therefore, obtained T-DNA insertion lines in these *BGLUs*, to test whether the lack of these BGLUs leads to altered ammonium sensitivity in our hypocotyl elongation test. In these lines, individual *BGLU* transcripts were partially (*BGLU10*) or entirely absent (*BGLU11* and *BGLU18*) (Figure 8, A and B). Interestingly, all three T-DNA insertion lines showed increased susceptibility to high ammonium concentrations in the hypocotyl elongation assay, consistent with a lack in ammonium signaling (Figure 8C).

## Discussion

The activity of many nutrient transporters in plants is balanced by a complex network of phosphorylation and dephosphorylation (Saito and Uozumi, 2020). Since ammonium toxicity is detrimental for most plants, a phospho-regulation network acting on plant AMT1s provides fast and efficient means to avoid this toxicity. Furthermore, the nutrition of plants has to be adjusted to their physiological status and to any abiotic stresses for acclimatization to environmental conditions as mediated via phytohormone signaling, including the stress hormone ABA (Li et al., 2014; Liu and Von Wirén, 2017; Marino and Moran, 2019).

Phosphorylation at a conserved C-terminal threonine inactivates plant AMT1 transporters (Loqué et al., 2007; Neuhäuser et al., 2007). However, adjacent to this conserved C-terminal phosphorylation site, AMT1 C-termini harbor several additional nonconserved phosphorylation sites, potentially integrating diverse local and systemic signals with ammonium nutrition (Menz et al., 2016; Wu et al., 2019).

The calcineurin B-like (CBL)-binding protein kinase CIPK23 has been shown to regulate the uptake of ammonium, potassium, and nitrate by its potential to phosphorylate several important transporters for these nutrients (Xu et al., 2006; Ho et al., 2009; Straub et al., 2017). The CIPK23-dependent phosphorylation of AMT1 transporters at the conserved C-terminal threonine results in a total inhibition of the trimeric AMTs (Loqué et al., 2007; Neuhäuser et al., 2007). Reactivation of the transporters occurs only after complete dephosphorylation (Neuhäuser et al., 2007).

This dephosphorylation requires a phosphatase counterpart to CIPK23. Here, we have identified ABI1 as regulating AMT1 activity by direct dephosphorylation, in addition to CIPK23 binding and subsequent inactivation.

ABI1 belongs to the A-subfamily of PP2C phosphatases and is a well-known negative regulator of the ABA response (Rodriguez, 1998; Schweighofer et al., 2004). Abiotic stress increases the intracellular ABA concentration, which causes the formation of PYL/ABA/ABI1 complexes and finally leads to ABI1 degradation (Park et al., 2009; Umezawa et al., 2009; Yin et al., 2009). Our data suggest that this complex formation is directly integrated into AMT1 regulation, since plants respond to an ammonium shock by an ABA increase—a mechanism previously established as being a signal of other abiotic stresses (Ma et al., 2018; Chen et al., 2020). A short exposure to high external ammonium concentrations is sufficient to provoke a fast and strong ABA increase in the plant roots (Figure 7A). Identification of the ammonium over-sensitive mutant AMOS1/EGY1 had previously predicted a connection between ammonium toxicity and ABA signaling (Li et al., 2012). Ammonium has also been implicated in ABA accumulation and root-to-shoot ABA translocation (Peuke et al., 1998; Li et al., 2012; Ding et al., 2016; Liu and Von Wirén, 2017). By mimicking this internal ABA increase, the external supply of ABA reduces ammonium uptake to a similar extent as the knockdown of *ABI1* (Figures 1, B and 7, B). Therefore, the external supply of ABA might lead to the direct inactivation or degradation of ABI1. The internal ABA increase occurs relative rapidly at 2.5 h after the 5-min ammonium shock. This is coherent with the expression increase of *BGLU* genes involved in ABA de-glycosylation (Figure 7C). De-conjugation of glucose from the ABA-glycosyl-ester not only increases its bioactivity, but also changes its subcellular localization from the ER and vacuole to the cytosol (Dietz et al., 2000; Kim et al., 2013). BGLU10 and BGLU11 are involved in activating ABA from the vacuole, whereas their homolog BGLU18 mediates ABA de-glycosylation in the ER (Xu et al., 2012; Ma et al., 2018). Their gene expression increases after the ammonium shock. Similarly, a fast increase in free ABA and a decrease of ABA-glycosyl-ester was found in wheat plants resupplied with nitrate. This deconjugation was mediated by a fast transcriptional increase of TaBG1 (Wang et al., 2020). Accordingly, the *bglu10*, *bglu11*, and *bglu18* lines, which partially miss the ability to deconjugate the glycosyl-ester, indeed showed a higher susceptibility to toxic ammonium conditions (Figure 8).

As shown previously, we have confirmed that external ABA application increases the interaction of ABI1 with almost all PYL proteins (Supplemental Figure S7) (Ma et al., 2009; Park et al., 2009; Yin et al., 2009; Nishimura et al., 2010). Therefore, we propose that an excessive external supply of ammonium will trigger ABA de-glycosylation in the vacuole and ER, which in turn leads to PYL/ABA/ABI1 complex formation and AMT1 inactivation by phosphorylation.

We have identified ABI1 as a possible AMT1 regulator in an initial screen for reduced ammonium susceptibility during hypocotyl elongation growth. The screen has led to the discovery of the knockdown line *abi1-2* (Supplemental Figure S1, a and b), which shows increased tolerance to toxic MeA and ammonium in the hypocotyl elongation assay (Figure 1;Supplemental Figure S2, a and b). Whereas the hydroponic growth of the *abi1-2* plants under standard conditions is indistinguishable from that of WT plants, the *abi1-2* plants exhibited reduced nitrogen in the shoot (Figure 2). This suggests a possible direct effect of ABI1 on nitrogen uptake.

The increased hypocotyl elongation in the dark was reversed in complementation lines, which mimicked the WT phenotype (Figure 1). We conducted an ammonium uptake assay in order to create a link between hypocotyl growth and uptake of ammonium. This short-term uptake experiment with 0.5- and 5-mM ^15^N ammonium led to the observation of reduced ammonium uptake by *abi1-2* plants was most pronounced in high-affinity uptake conditions (0.5 mM ^15^${\text{NH}}_{4}^{+}$) (Figure 3). This implies an involvement of AMT1-mediated high-affinity ammonium transport in the effects of the *abi1-2* mutation. In low-affinity conditions, apoplastic flow is most likely to mask this effect on AMT1 activity.

Western blot using an AMT1;2 antibody (Supplemental Figure S3), expression analysis (Supplemental Figure S5), and confocal laser scanning microscopy (Supplemental Figure S6) have excluded that lower AMT1 protein abundance, *AMT1* expression or altered subcellular localization away from the plasma membrane are responsible explanations for the reduced ammonium uptake in the *abi1-2* line. Therefore, we concluded that the AMT1 phosphorylation status should be directly affected. To confirm this hypothesis, we first tested for an interaction of ABI1 with CIPK23, and with the AMT1 transporters, a prerequisite for phosphorylation. Interestingly, protein interaction studies involving the use of endogenous promoters in planta, split ubiquitin in yeast, and BiFC in *X.laevis* oocytes, all consistently revealed that ABI1 interacts with CIPK23, in addition with both AMT1;1 and AMT1;2 (Figure 4). We, therefore, hypothesize an additive effect of ABI1 on ammonium uptake via the inhibition of CIPK23 and the direct dephosphorylation of transporters. There was no interaction with AMT1;3, which as well was not found to be regulated by CIPK23 (Straub et al., 2017). In an attempt to restrict the possible ABI1-dephosphorylation sites in the AMT1 C-termini, we tested the interaction of ABI1 with deletion mutants of the AMT1 C-termini. For AMT1 mutants in which only the conserved threonine dephosphorylation site remained, we still observed interaction between transporters and the phosphatase (Figure 5). This finding is in agreement with the idea that ABI1 interacts with and possibly dephosphorylates this conserved phosphorylation site.

The phosphorylation status of the AMT1s matched well with the data from ^15^N ammonium uptake. Under N starvation, the *abi1-2* plants showed the highest amount of AMT1 phosphorylation (Figure 6A), resulting in reduced ammonium uptake, in comparison with all other lines (Figure 3). In contrast to the first time point, all lines exhibited an increase in phosphorylation at the second timepoint at 2.5 h after ammonium shock (Figure 6). As expected, this also affected ammonium uptake, which was generally reduced after ammonium shock, but showed no significant differences between the lines. An additional 5 h were required until the phosphorylation pattern of the four lines resembled the result following initial N starvation (Figure 6D). The phosphorylation status after 5 h showed that dephosphorylation of the AMT1s occurred faster in the “ABI1 overexpressing” complementation lines than in the WT, whereas it was absent in the *abi1-2* plants (Figure 6B;Supplemental Figure S4). Additional to demonstrating the regulation of CIPK23, these data agree with a direct AMT1 dephosphorylation function of ABI1. Still, direct dephosphorylation of AMT1s by ABI1 remains to be tested.

**Figure 9:**
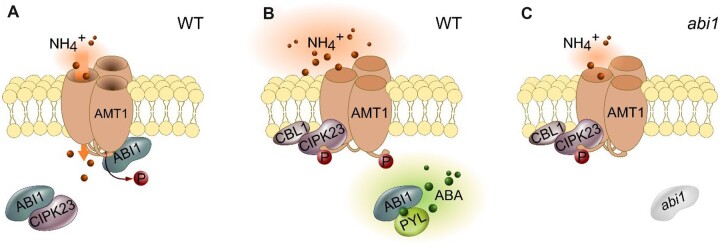
Proposed model of ammonium uptake regulation by ABI1 and CIPK23. A, Under ample ammonium conditions, ABI1 will bind and inactivate CIPK23 while binding and activating AMT1;1 and AMT1;2. B, Under conditions of ammonium toxicity and other types of abiotic stress, plants produce active ABA. ABA receptors capture ABI1 and form a PYL/ABA/ABI1 complex. CIPK23 becomes released and active, phosphorylating and inactivating the AMT1 enabling the plant to avoid ammonium uptake and toxicity. C, Higher AMT1 phosphorylation and reduced ammonium uptake capacity, even under low ammonium conditions in *abi1-2* knockdown plants.

We, therefore, conclude that, under low ammonium, nonstressed conditions, ABI1 binds to and inactivates CIPK23, whereas it simultaneously binds and keeps AMT1s active (Figure 9A). High external ammonium conditions stimulate a fast and strong ABA increase in the plant roots. This ABA is sensed by PYL ABA receptors, which bind and inactivate ABI1 (Rodriguez et al., 2019). ABI1 inactivation liberates CIPK23, which in turn binds to and phosphorylates AMT1. This phosphorylation then shuts down AMT1 transport activity to reduce the ammonium toxicity effects (Figure 9B). In the *abi1-2* knockdown plants, CIPK23 remains hyperactive, leading to a reduced ammonium susceptibility and higher AMT1 phosphorylation, plus reduced ammonium uptake, even under control conditions (Figure 9C). On the one hand, plants thus sense ammonium toxicity in the same way as other abiotic stresses and use the ABA signaling cascade to integrate this stress with their ammonium nutrition. On the other hand, nutritional acclimatization as a response to abiotic stresses is probably also mediated by ABA suggesting that ABA signaling limits nutrient (ammonium) uptake under abiotic stress. This might be a general mechanism because nutrient demands are lower when growth is impaired by stress. Investigations into whether other stresses have an effect on ammonium uptake and whether the ABA/ABI1-dependent CIPK23 regulation network also influences the transport of other ions, such as nitrate and potassium, should be of future interest.

## Materials and methods

### Plant material

WT Arabidopsis (*A.thaliana*) Col-0, a knockdown mutant of *ABI1* (AT4G26,080) *abi1-2* (NASC Nr.: N655,606; SALK_072,009C), complementation lines generated by agrobacterium-mediated transformation of *abi1-2* with a WT *ABI1* coding sequence having the 1,000-bp promoter region and previously used AMT/CIPK23 transgenic plants (Straub et al., 2017) were used in all experiments. For *BGUL10* (At4g27,830), *BGLU11* (At1g02,850), and *BGLU18* (At1g52,400) the T-DNA insertion lines *bglu10* (NASC Nr.: N868866; SALK_101,672), *bglu11* (NASC Nr.: N860,216; SAIL_62_D08), and *bglu18* (NASC Nr.: N666452; SALK_075,731) were used. Additional 73 T-DNA insertion lines close to or in the coding region of phosphatases highly expressed in the root were used in the initial screen for AMT1 activators (Supplemental Table S1).

### Generation of complementation lines

A genomic fragment covering the 1,000-bp promoter sequence upstream of the start codon and the open reading frame of *ABI1* were cloned into pTbar vector yielding the transformation vector *pTbar pABI1::ABI1*. Homozygous *abi1-2* plants were transformed with the floral-dip method (Clough and Bent, 1998) by using the *Agrobacterium tumefaciens* strain GV3103. Plants were propagated for three generations and two independent homozygous complementation lines were obtained. Selection was performed on 1/2 MS media supplemented with 4 µM L-methionine sulfoximine. Gene expression of *ABI1* was verified by qPCR.

### Ammonium uptake assay

A hydroponic system was used to grow the plants for 6 weeks in quarter-strength Hoagland solution at pH 6 with 1-mM NH_4_NO_3_. The controlled environmental conditions were: 8 h short day (light 200 µmol m^−2^ s^−1^) with a temperature between 20°C and 22°C and a humidity range of 50%–60%. After 6 weeks, plants were transferred to 1/4 Hoagland media lacking nitrogen and starved for 4 days. Afterward, the plants were transferred to 1/4 Hoagland media supplemented with 0.25 and 2.5 mM (^15^NH_4_)_2_SO_4_ for 10 min. Roots used for ^15^N-measurements were washed twice with 1 mM CaSO_4_, briefly dried with a paper towel, frozen, and freeze-dried. Plants for the second timepoint were placed in shocking solution containing high concentrations of (NH_4_)_2_SO_4_ for 5 min and harvested after uptake in the same way as that described above at 2.5 h after the beginning of the shock. Depending on the experiment, either 10- or 20-mM ammonium was used as an ammonium shock treatment as indicated in the legends of the corresponding figures. Both concentrations induced comparable effects in independent experiments. The dried plant material was ground and 0.5 mg was taken to measure the δ^15/14^N ratio by using isotope ratio mass spectrometry (Delta plus Advantage, Thermo Fisher Scientific, Waltham, MA, USA, coupled to an Eurovector elemental analyzer EA 3000).

### Gene expression analysis

Samples were taken at each harvesting timepoint of the ammonium uptake assay and immediately frozen in liquid nitrogen. Afterward, they were ground using mortar and pestle and five plants were pooled as one biological sample. Total RNA was extracted with the innuPREP Plant Extraction kit from Analytik Jena and the concentration was measured using a NanoDrop2000 spectrophotometer (Thermo Fisher Scientific Inc., Waltham, MA, USA). Reverse transcription was performed with the QuantiTect Reverse Transcription Kit (Qiagen GmbH, Hilden, Germany) according to the manufacturer’s manual. The qPCR was set up with the GreenMasterMix (GENAXXON bioscience, Ulm. Germany) and a C1000 Thermo Cycler attached to a CFX 384 real-time system (Bio-Rad Laboratories Ltd., Watford, UK). Each reaction volume of 15 μL contained 10 ng cDNA. Relative expression was calculated using the ΔΔCT method with UBQ10 and SAND as reference genes. The gene numbers and the primers used are shown in Supplemental Table S2.

### Yeast two-hybrid interaction assay

The coding sequences of *ABI1*, *AMT1;1*, *AMT1,2*, *CIPK23*, and all *PRY/PYL* genes were amplified from cDNA and cloned into pCR Blunt II-TOPO vector (Thermo Fisher Scientific, Waltham, MA, USA) followed by amplification with restriction site-containing primers and subcloning into the corresponding vectors as described below. The open reading frame of *ABI1* was cloned into pPR3-N, whereas *AMT1;1*, *AMT1;2*, their C-terminal mutants, and *CIPK23* were ligated into pBT3-C, both vectors being part of the DUALmembrane Starter Kit (Dualsystems Biotech AG, Schlieren, Switzerland). The *PRY/PYL* genes were also cloned into pPR3-N. Combinations of both vectors were transformed into yeast strain NMY51 by the lithium acetate method (Gietz and Schiestl, 2007). Positive transformants were selected on solid synthetic defined (SD)–Trp–Leu medium. Colonies for the assay were picked and grown overnight in liquid SD–Trp–Leu at 28°C. Afterward, they were washed three times with water and adjusted to an optical density (OD)_595_ = 10. Three 5× dilutions (OD_595_ = 10, 2, and 0.4) of yeast suspension in a volume of 10 µL were spotted onto SD plates without -Trp–Leu–Ade, -Trp–Leu–His, or -Trp–Leu–Ade–His; for ABI1/PYL interaction only a dilution with an OD_595_ = 2 was spotted but plates were further supplemented with 10-µM ABA. Yeast was grown for 5 days at 28°C and were subsequently covered with an X-Gal overlay. The mutants *AMT1;1ΔS488-V501* and *AMT1;1ΔT497-V501* were generated by site-directed mutagenesis PCR resulting in a deleted C-terminus followed by a stop codon.

### Split-YFP interaction *X. laevis* oocytes and in planta

Oocytes were ordered at EcoCyte Bioscience (Castrop-Rauxel, Germany), pre-sorted again and injected with 50 nL of cRNA. Total cRNA amount was 1 µg containing a mix (500 ng + 500 ng) of the respective Gene-YFPpart fusions. Oocytes were kept in ND96 for 4 days at 18°C and then analysed by confocal microscopy as described below. The in planta interaction between ABI1 and AMT1;1/AMT1;2/CIPK23 was tested as previously described (Straub et al., 2017). With regard to the design of the fusion construct, the YFP molecule was split between amino acids 153 and 154 and these parts were inserted into the pTkan^+^ and pTbar vectors to give the pTkan^+^YN 153stop and pTbar YC stop vectors. The vectors containing the AMT1s and CIPK23 were available in our laboratory. The respective promoter:gene fusion for ABI1 was cloned into the two vectors by using appropriate single cutting enzymes in the multiple cloning site. The various plasmid combinations were transformed into *A.thaliana* Col-0 plants as described above. Transgenic plants were analysed by using a LSM700_ZEN_2010 microscope (Zeiss, Germany) using respective settings (pinhole = 70 µm; master gain = 955; digital gain = 1; digital offset = 0; Laser = 488 nm, 2.5%) for YFP (filter = 505–1,000 nm) or GFP (filter = 500–1,000 nm). Laser intensity, pinhole and detection range stayed unchanged and were similar for all pictures. Background in the images might increase depending on the position of the oocyte in the petri dish.

### Protein extraction and immunoblotting

Total protein was extracted from frozen root tissue by using cold extraction buffer (100 mM NaCl, 50 mM Tris–HCl (pH = 7.5), 0.5% (v/v) Triton X-100, 10-mM β-mercaptoethanol) supplemented with PhosSTOP (Roche, Basel, Switzerland) and Complete Protease Inhibitor Cocktail (Roche, Basel) according to the manufacturer’s manual. Samples were then centrifuged and the protein concentration in the collected supernatants was measured using the Bradford assay. Proteins in amounts of 10 µg were denatured in Laemmli sample buffer (62.5-mM TRIS pH 6.8, 2% (w/v) sodium dodecyl sulfate (SDS), 10% (v/v) glycerol, 5% (v/v) β-mercaptoethanol, 0.001% (w/v) bromphenol-blue) for 10 min at 50°C. Subsequently, samples were loaded onto a 12% acrylamide SDS gel and separated by electrophoresis. Transfer to a nitrocellulose membrane was performed by semi-dry blotting with standard Bjerrum Schafer-Nielsen buffer (Bio-Rad Laboratories GmbH, Feldkirchen). Membranes were blocked using TBS-T containing 1% (w/v) casein hydrolysate for 1 h followed by overnight incubation in blocking solution and primary antibody (IgG, rabbit, dilution 1:1,000; BioGenes, Berlin, Germany). The custom-made antibody detected the phosphorylated conserved threonine in AMT1 (CG-NleD-Nle-pT-RHGGFA-amide, Figure 3B). As a control an commercially available AMT1;2 antibody was used (rabbit polyclonal, anti-AMT1;2, 1:1,000, PhytoAB Inc.; Ganz et al. 2020) After three washing steps, the membrane was incubated for 1 h in TBS-T supplemented with the secondary antibody (polyclonal IgG, goat, conjugated to horseradish peroxidase, dilution 1:5,000; Roth, Karlsruhe). The membrane was subsequently washed with TBS and treated with ECL SuperSignal West Dura solution (Thermo Fisher Scientific, Waltham, MA, USA). Signals were detected on an Odyssey Fc imager (Li-COR Biotechnology; Bad Homburg). The intensity of the bands was measured using ImageJ software. For a loading control, the membrane was incubated for 2 min in 0.1% (w/v) Ponceau S in 5% (v/v) acetic acid. Afterwards, the membrane was washed twice in distilled H_2_O.

### Hypocotyl elongation assay

Sterilized Arabidopsis seeds were distributed on square petri dishes containing MQ water supplemented with 20 g L^−1^ agar and 40-mM MeA, 15 and 20 mM NH_4_Cl, or 0-mM MeA/NH_4_Cl. The plates were incubated at 4°C for 2 days. Germination was induced by incubation in a Percival AR-66L (150 μmol m^−2^s^−1^, Philips F17T8/TL841 17W) for 1 day, followed by 5 days incubation in the dark. Hypocotyl length was measured using ImageJ software and the growth reduction of the *abi1-2* mutant and the complementation lines was normalized to control conditions and/or Col-0’s hypocotyl length. The experiment was repeated 3 times; approximately 30 hypocotyls were measured per treatment, line, and repetition.

### ABA measurements

Frozen Arabidopsis roots were ground to a fine powder with liquid nitrogen and 1-g plant material was treated with 80% (v/v) methanol. Supernatants were collected after centrifugation and cleaned by membrane filtration (Chromafil O-20/15 MS). Samples were then analyzed by ultra-high performance liquid chromatography-mass spectrometry (UHPLC-MS) on a Velos LTQ System (Thermo Fisher Scientific, Waltham, MA, USA) as described in more detail by Moradtalab and co-workers (Moradtalab et al., 2018).

### Statistics

At least three repetitions were conducted in all experiments. The numbers of the replicates in one experiment are given in the figure legends. Statistical significance was tested by an analysis of variance (ANOVA) followed by Tukey’s post-hoc test. Significant differences are indicated by capital or small letters (*P* < 0.05). When the ANOVA was followed by a pairwise comparison, significant differences are indicated by ^***^P <  0.001.

## Accession numbers

Sequence data from this article can be found in the GenBank/EMBL data libraries under accession numbers: AT4G26080 (*ABI1*), AT4G13510 (*AMT1;1*), AT1G64780 (*AMT1;2*), AT3G24300 (*AMT1;3*), AT4G27830 (*BGLU10*), AT1G02850 (*BGLU11*), AT1G52400 (*BGLU18*), and AT1G30270 (*CIPK23*).

## Supplemental data

The following materials are available in the online version of this article.

**Supplemental Figure S1.** Genotyping of the *abi1-2* and complementation (OX) lines.

**Supplemental Figure S2.** Reduced ammonium toxicity in the *abi1-2* knockdown line.

**Supplemental Figure S3.** Unchanged AMT1;2 protein abundance in the analyzed plant lines.

**Supplemental Figure S4.** Quantification of ABI1 effects on phosphorylation status of conserved AMT1 C termini.

**Supplemental Figure S5.** *AMT1* expression is not reduced in *abi1-2* mutants.

**Supplemental Figure S6.** Localization of AMT1;1 and AMT1;2 is unaffected on a Col-0 or *abi1-2* background.

**Supplemental Figure S7.** Protein interaction of ABI1 with protein family of PYR/PYL ABA receptors.

**Supplemental Table S1.** T-DNA insertion lines used in the initial screen.

**Supplemental Table S2.** qPCR and genotyping primers used in this study.

## Supplementary Material

kiac315_Supplementary_Data

## Notes

P.G., R.P-.M., T.I., N.S., U.L., and B.N. designed the experiments. P.G., R.P-.M., T. I., N.S., J. M., N.M., and B.N. performed the experiments. P.G., R.P-.M., T.I., N.S., and B.N. evaluated and interpreted the data. P.G, T.S., and B.N. prepared the figures. P.G. and B.N. wrote the manuscript. P.G., U.L., N.S., T.S., and B.N. revised the manuscript. B.N. obtained the funding and is the corresponding author.

The author responsible for distribution of materials integral to the findings presented in this article in accordance with the policy described in the Instructions for Authors (https://academic.oup.com/plphys/pages/general-instructions) is Benjamin Neuhäuser (benjamin.neuhaeuser@uni-hohenheim.de).
