# Supplementary material for: Abscisic acid influences ammonium transport via regulation of kinase CIPK23 and ammonium transporters
Source: Plant Physiol. 2022 Jun 28;190(2):1275–88. doi: 10.1093/plphys/kiac315 (PMC9516733; doi:10.1093/plphys/kiac315)
Supplement: kiac315_Supplementary_Data [file kiac315_supplementary_data.pdf]

Short Title:

ABA-dependent ammonium transporter regulation

Author for Contact:

Benjamin Neuhäuser (Email: benjamin.neuhaeuser@uni-hohenheim.de)

Title:

Abscisic acid influences ammonium transport via regulation of kinase CIPK23 and ammonium transporters

Authors:

Pascal Ganz[1], Romano Porras-Murillo[1], Toyosi Ijato[1], Jochen Menz[1], Tatsiana Straub[1], Nils Stührwohldt[2], Narges Moradtalab[1], Uwe Ludewig[1] and Benjamin Neuhäuser[1]

Supplementary Information

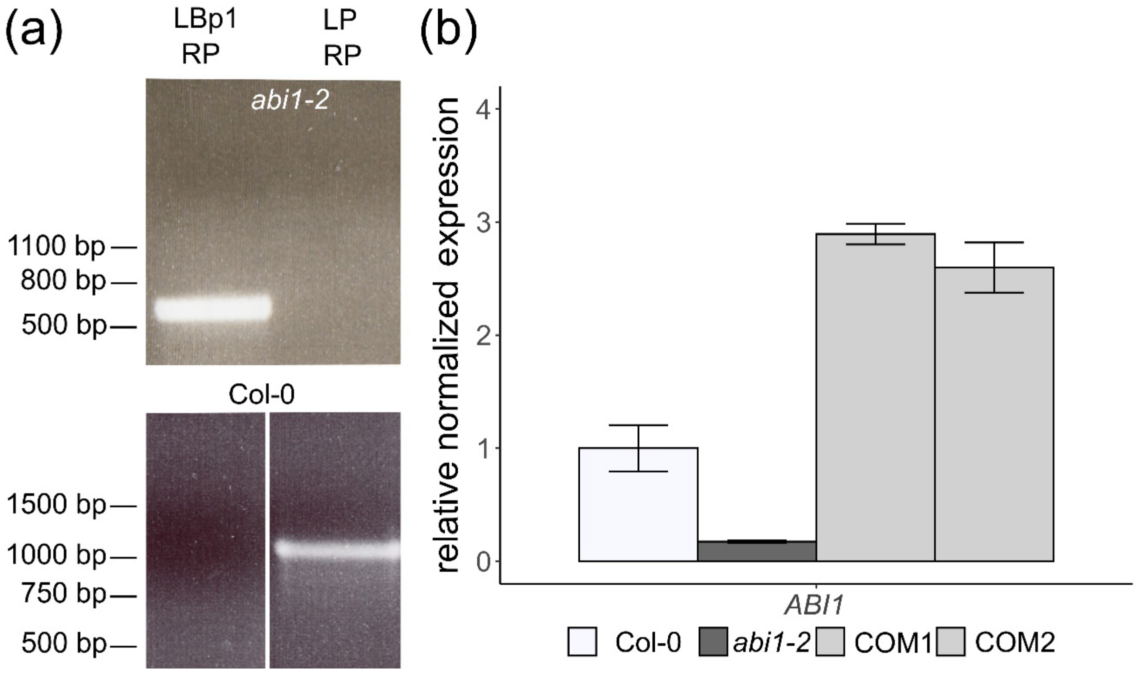

Supplemental Figure S1. Genotyping of the *abi1-2* and complementation (OX) lines. (a) Genotyping of the *abi1-2* (NASC Nr.: N655606; SALK_072009C) line revealing a homozygous T-DNA insertion in *ABI1* (AT4G26080), upper part. Col-0 negative control, lower part. LBp1 = Left boarder primer; RP = right primer; LP = left primer (please see Tab. S2) **(b)** Relative normalized *ABI1* expression in roots of Col-0, *abi1-2* and the complementation (endogenous promoter) line plants. Data are shown as mean ±SEM (n = 3).

(a)

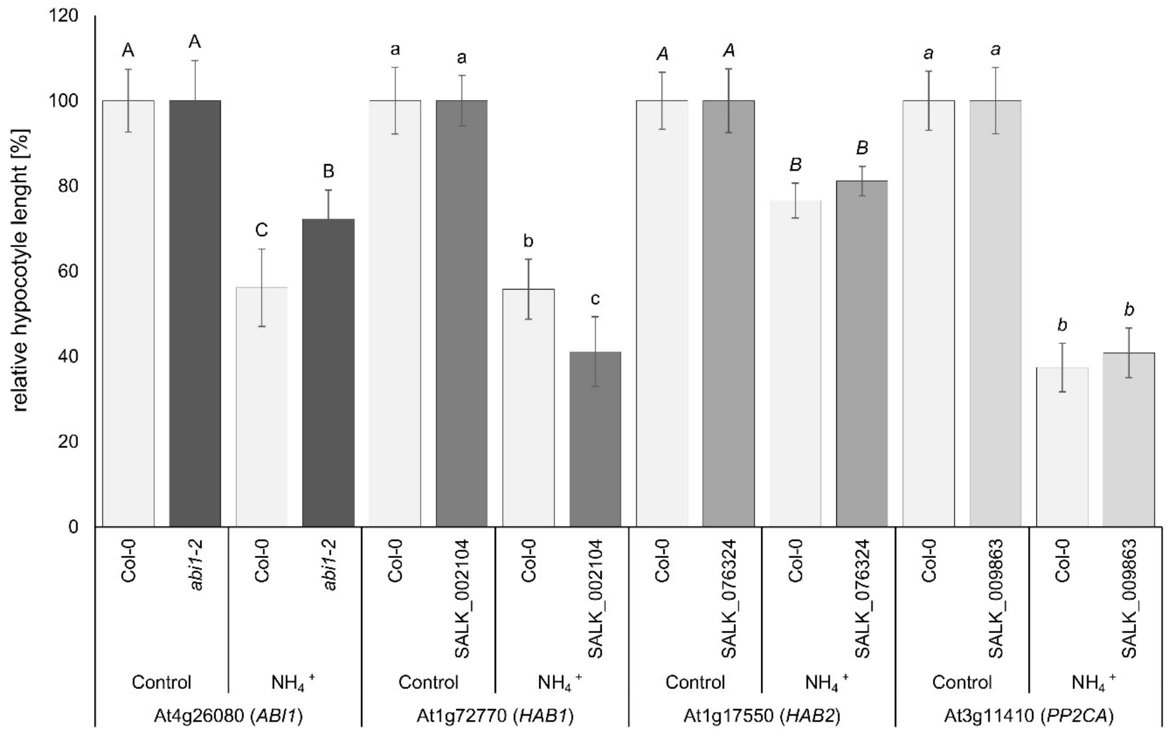

(b)

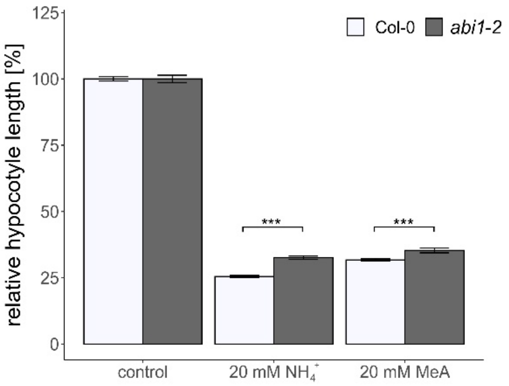

Supplemental Figure S2. Reduced ammonium toxicity in the *abi1-2* knockdown line. (a) Relative normalized hypocotyl length of etiolated seedlings of PP2C class A phosphatases. Data from the original screen normalized to the control treatment. Data are shown as means ±SEM. Statistical significance was tested by an ANOVA followed by Tukey's post-hoc test. Significant differences are indicated by capital or small letters ($n \geq 20$; $p < 0.01$). **(b)** Relative normalized hypocotyl length of etiolated Col-0 and *abi1-2* seedlings grown on control media or media containing 20 mM NH_4^+/methylammonium. Data are shown as means normalized to the control ±SEM. Statistical significance was tested by an ANOVA followed by a pairwise comparison, significant differences are indicated by *** ($n \geq 200$; $p < 0.001$).

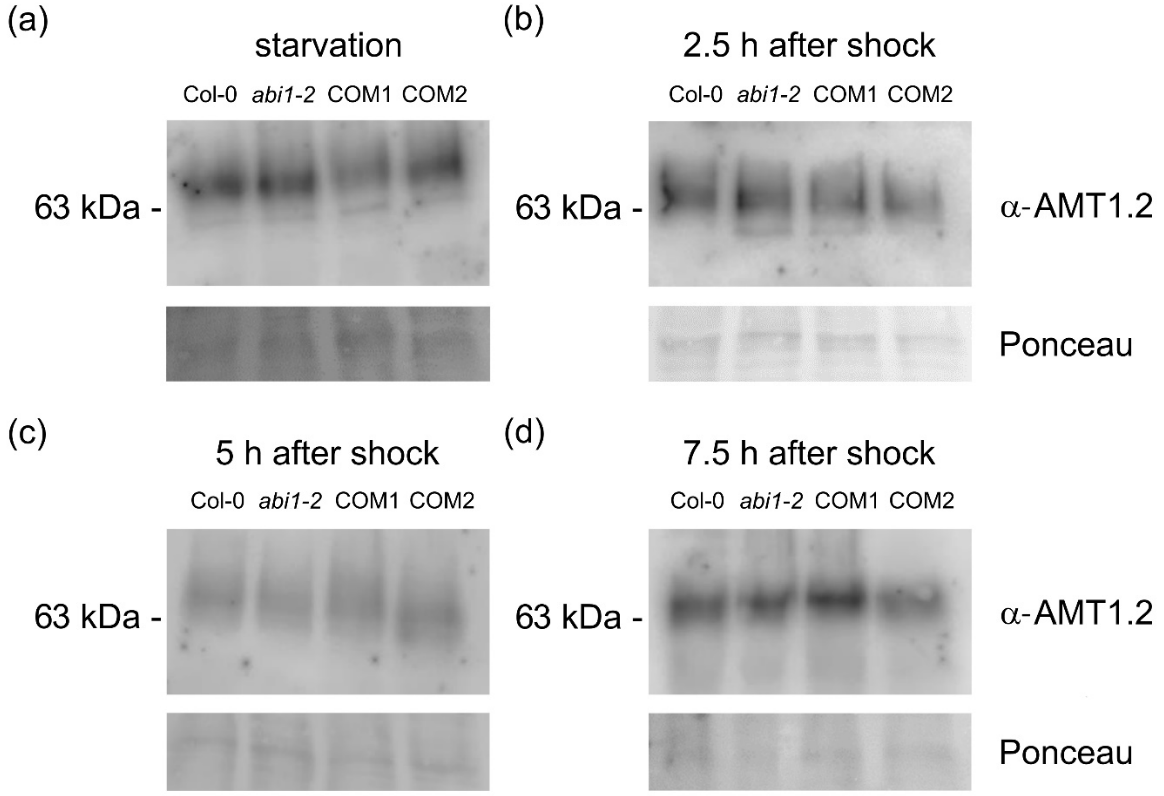

Supplemental Figure S3. Unchanged AMT1;2 protein abundance in the analysed plant lines. Protein gel blot analysis of total root protein extract from Col-0, *abi1-2* and complementation lines (COM1 and COM2) by using an AMT1;2 specific antibody detecting total AMT1;2 protein abundance after nitrogen starvation for four days or after a nitrogen shock followed by 2.5 h, 5 h or 7.5 h of renewed nitrogen starvation. Upper part shows detection of AMT1,2 and lower part shows loading controls stained with Ponceau red.

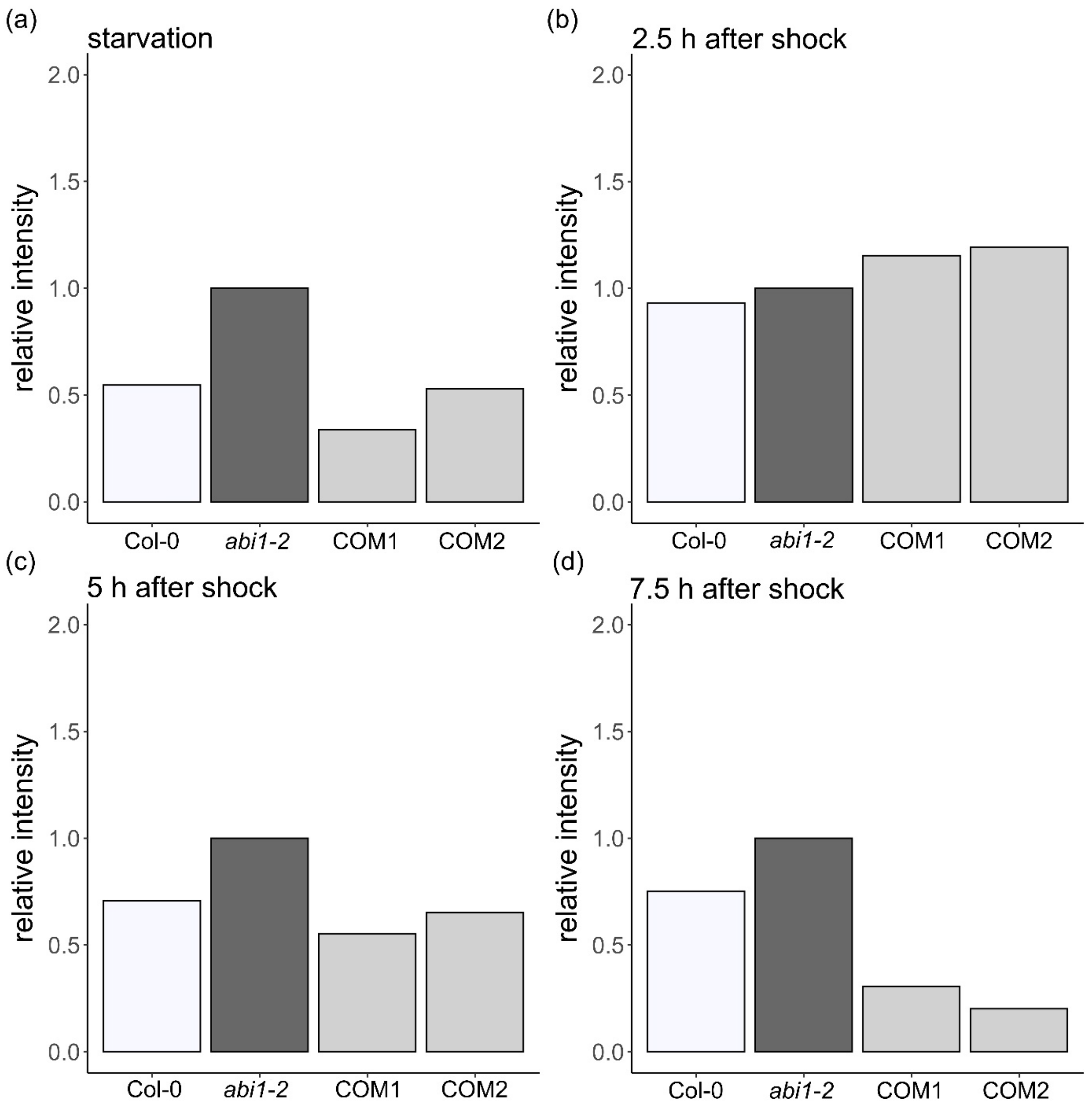

Supplemental Figure S4. Quantification of ABI1 effects on phosphorylation status of conserved AMT1 C-termini. Protein gel blot analysis of total root protein extract from Col-0, *abi1-2* and complementation line plants by using a phosphorylation-specific antibody detecting the conserved AMT1 C-terminus shown in Figure 3 was quantified by ImageJ. Analysis of the two other replicates yielded similar results.

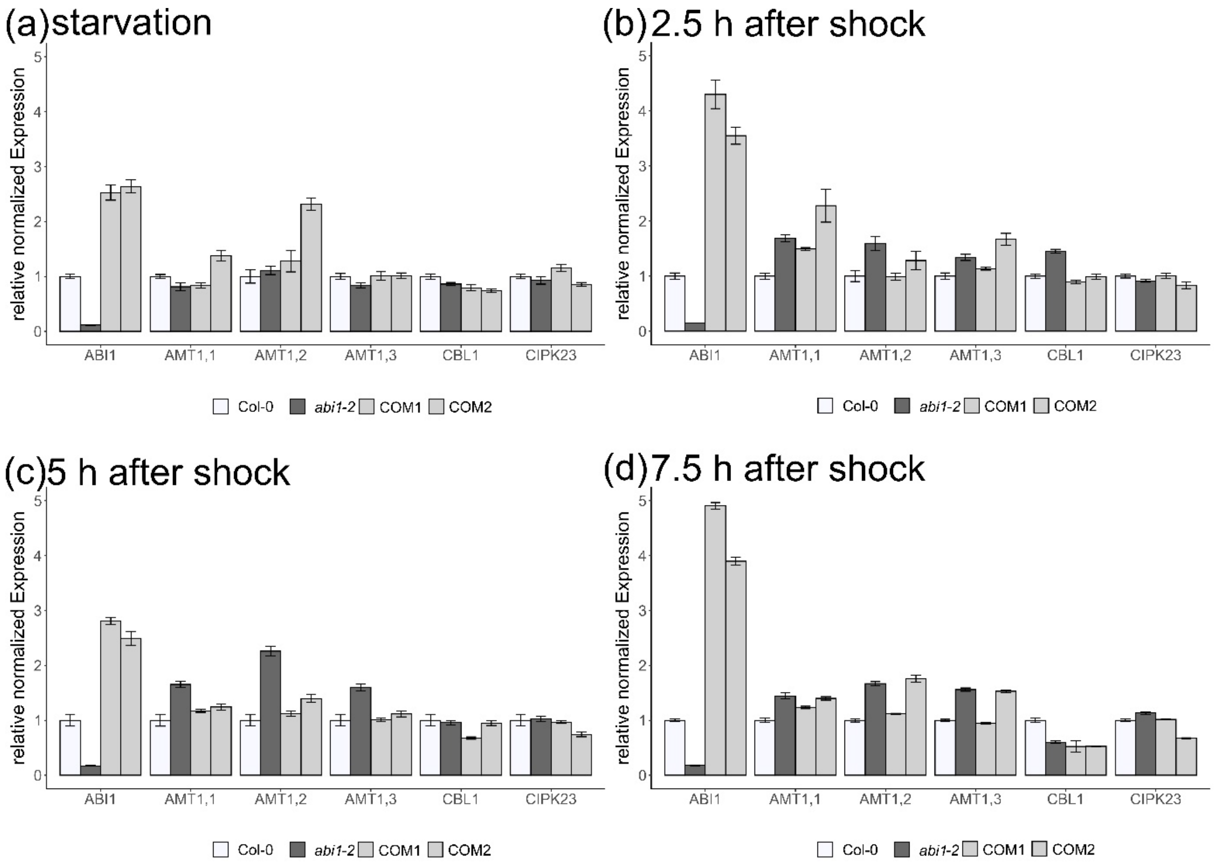

Supplemental Figure S5. ***AMT1*** **expression is not reduced in** ***abi1-2*** **mutants.** Gene expression for *AMT1*s and their regulatory genes *CBL1, CIPK23* and *ABI1* in roots of 6-week-old Col-0, *abi1-2* and the two complementation lines. After starvation **(a)** and after 2.5 h **(b)**, 5h **(c)** and 7.5 h **(d)** of starvation after a 5-min ammonium shock. Data are shown as mean ±SEM (n = 3)

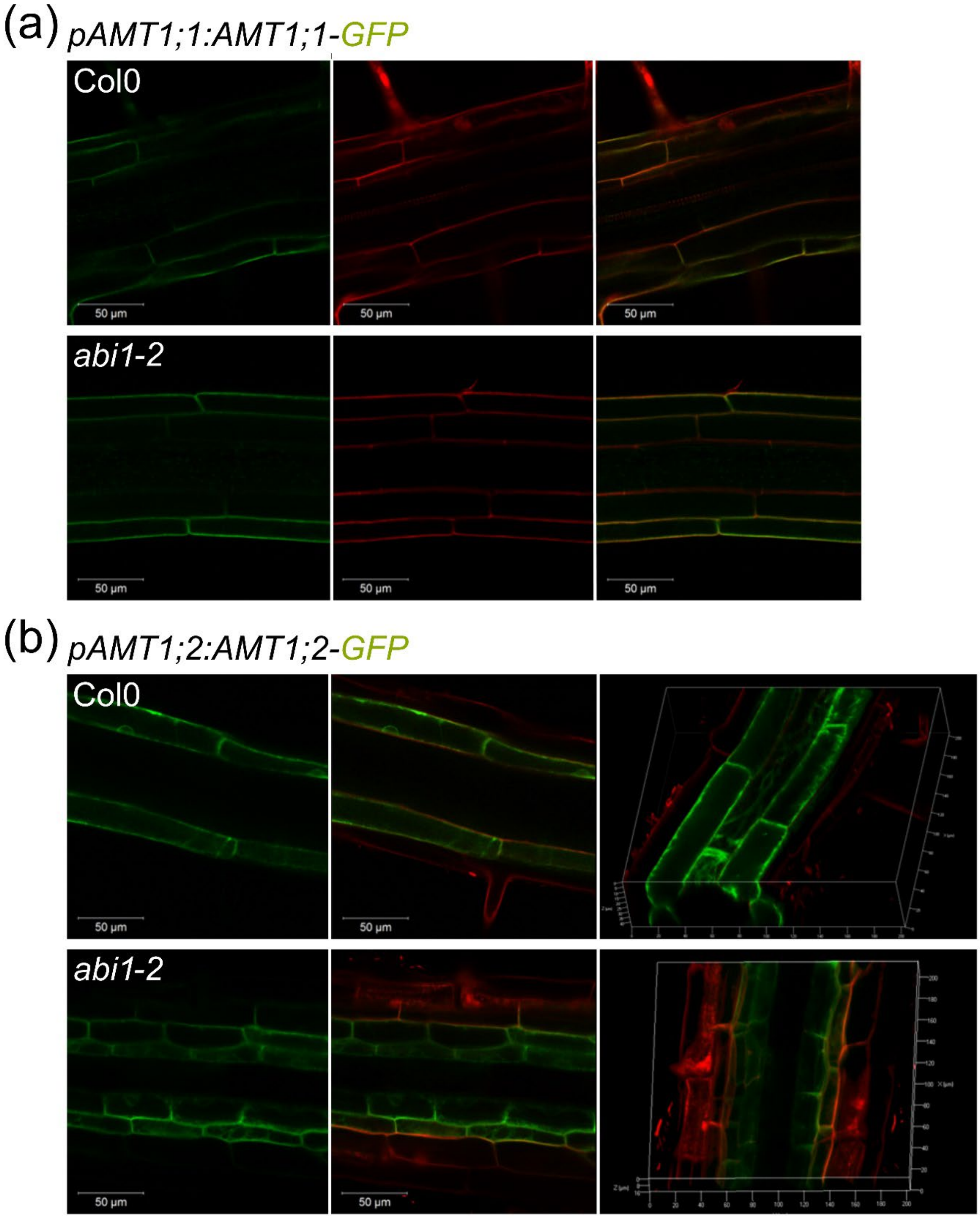

Supplemental Figure S6. Localization of AMT1;1 and AMT1;2 is unaffected on a Col-0 or *abi1-2* background. Fusion construct of pAMT1::AMT1-GFP and **(a)** AMT1;1 or **(b)** AMT1;2 expressed on a wild-type or *abi1-2* background; fluorescence was monitored by laser scanning confocal microscopy. In **(a)** from left to right: GFP fluorescence, propidium iodide, overlay. In **(b)** from left to right: GFP fluorescence, overlay with propidium iodide, 3D view.

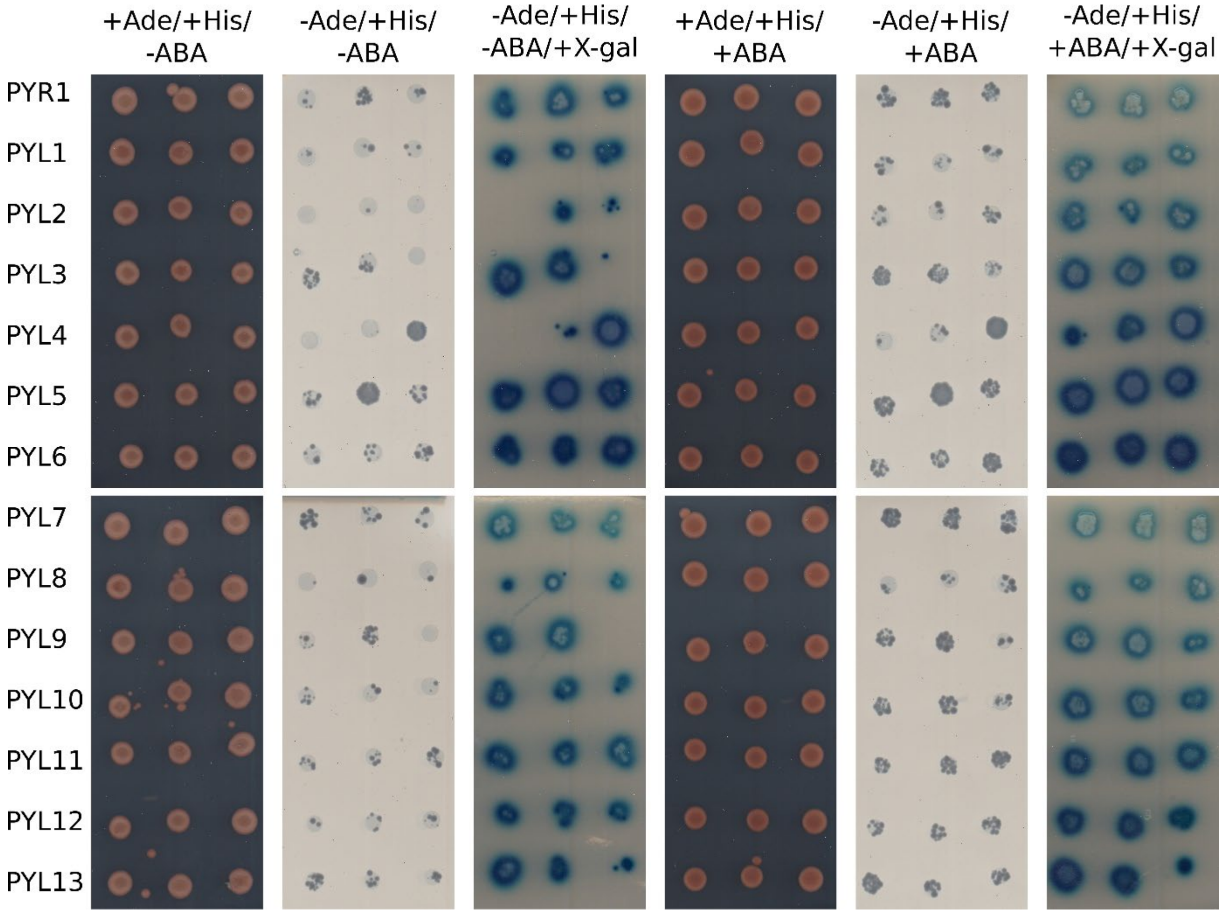

Supplemental Figure S7. Protein interaction of ABI1 with protein family of PYR/PYL ABA receptors. ABI1 is fused to the C-terminal part of ubiquitin, whereas the interaction partners are fused to the N-terminal part of ubiquitin. Growth on media without adenine and blue staining by X-Gal overlay indicates protein interaction. From left to right, control without ABA, media without adenine and ABA, X-Gal overlay of media without adenine and ABA, control with ABA, media without adenine and with ABA, X-Gal overlay of media without adenine and with ABA.

Supplemental Table S1. T-DNA insertion lines used in the initial screen. The number of the Arabidopsis stock centre (www.arabidopsis.info), the identification number of the T-DNA insertion line, the number of the gene associated with this insertion line, the gene family of the phosphatase and the name of the phosphatase are given.

NASC number	SALK number	AGI Code	Gene family	Gene name
307140	GK-065G03.02	At1g07160	PP2C	AtPP2C2
308984	GK-316H10.04	At4g27800	PP2C	AtPP2C57/AtPPH1
315124	GK-072G03.04	At1g10430	PP2A	AtPP2A1
653077	020722C	At4g11240	PP1	AtTOPP7
653085	021793C	At3g06270	PP2C	AtPP2C35
654391	128186C	At4g16580	PP2C	AtPP2C55
655403	029481C	At1g18030	PP2C	AtPP2C8
655606	072009C	At4g26080	PP2C	AtPP2C56/AtABI1
656121	005558C	At5g51760	PP2C	AtPP2C75
656211	016641C	At3g12620	PP2C	AtPP2C38
656223	017899C	At5g66720	PP2C	AtPP2C80
656230	019305C	At2g20630	PP2C	AtPP2C20/AtPPC3
656443	045433C	At3g05580	PP1	AtTOPP9
656660	076324C	At1g17550	PP2C	AtPP2C7/AtHAB2
656851	108282C	At5g59220	PP2C	AtPP2C78
657084	146020C	At4g31750	PP2C	AtPP2C59/AtWIN2
657124	151946C	At5g55260	PP4/PPX	AtPPX2
657178	025713C	At4g27800	PP2C	AtPP2C57/AtPPH1
658262	061302C	At3g16800	PP2C	AtPP2C41
658526	016135C	At3g17250	PP2C	AtPP2C43
659308	013178C	At1g69960	PP2A	AtPP2A5
659594	146162C	At2g34740	PP2C	AtPP2C28
659830	042824C	At5g27930	PP2C	AtPP2C73/AtPPC6
659855	048861C	At5g27930	PP2C	AtPP2C73/AtPPC6
659861	049725C	At5g55260	PP4/PPX-type	AtPPX2
660122	115494C	At5g02400	PP2C	AtPP2C66/AtPLL2
660233	143218C	At1g17550	PP2C	AtPP2C7/AtHAB2
661958	027747C	At3g06270	PP2C	AtPP2C35
662121	036920C	At3g12620	PP2C	AtPP2C38
662642	060018C	At1g07160	PP2C	AtPP2C2
663581	105978C	At2g20630	PP2C	AtPP2C20/AtPPC3
663774	117607C	At3g05580	PP1	AtTOPP9
664102	136570C	At1g43900	PP2C	AtPP2C11
664699	076309C	At4g26080	PP2C	AtPP2C56/AtABI1
665105	002104C	At1g72770	PP2C	AtPP2C16/AtHAB1
666967	106575C	At2g34740	PP2C	AtPP2C28
667683	003931C	At5g06750	PP2C	AtPP2C68
667710	006360C	At1g78200	PP2C	AtPP2C17
667834	015078C	At3g62260	PP2C	AtPP2C49
668879	113717C	At4g33500	PP2C	AtPP2C62
668957	122669C	At5g02400	PP2C	AtPP2C66/AtPLL2
669012	127487C	At4g28400	PP2C	AtPP2C58
669372	011182C	At1g69850	PP2C	AtPP2C75
669553	033011C	At2g29380	PP2C	AtPP2C24
670093	123000C	At3g09400	PP2C	AtPP2C36/AtPLL3
671069	003944C	At1g22280	PP2C	AtPP2C9
671261	014358C	At4g33500	PP2C	AtPP2C62
672391	025675C	At3g62260	PP2C	AtPP2C49
672967	095052C	At5g51760	PP2C	AtPP2C75
673054	100800C	At1g78200	PP2C	AtPP2C17
673072	102599C	At1g59830	PP2A	AtPP2A2
673122	106442C	At1g09160	PP2C	AtPP2C5
673629	002822C	At1g09160	PP2C	AtPP2C5
673670	005240C	At2g25620	PP2C	AtPP2C22
673778	009279C	At1g03590	PP2C	AtPP2C1
673798	010368C	At5g53140	PP2C	AtPP2C76
674413	034856C	At5g59160	PP1	AtTOPP2
675718	072696C	At1g69850	PP2C	AtPP2C75
676774	123840C	At1g47380	PP2C	AtPP2C12
676924	130437C	At1g07630	PP2C	AtPP2C4/AtPLL5
678094	009863C	At3g11410	PP2C	AtPP2C37/AtPP2CA
678792	088465C	At3g17250	PP2C	AtPP2C43
679192	143298C	At4g33920	PP2C	AtPP2C63
679596	044162C	At1g07630	PP2C	AtPP2C4/AtPLL5
823387	SAIL_552_G10	At1g22280	PP2C	AtPP2C9
833138	SAIL_742_A05	At3g02750	PP2C	AtPP2C33
840122	SAIL_891_C01	At3g23360	PP2C	AtPP2C44
850628	WiscDsLox293-296invD18	At2g39840	PP1	AtTOPP4
851819	WiscDsLox339E05	At4g31750	PP2C	AtPP2C59/AtWIN2
873621	SAIL_378_F05	At1g59830	PP2A	AtPP2A2
878169	SAIL_1151_F04	At2g29400	PP1	AtTOPP1
903539	WiscDsLoxHs037_11C	At1g64040	PP1	AtTOPP3
911811	WiscDsLoxHs124_01C	At1g68410	PP2C	AtPP2C15

Supplemental Table S2: qPCR and genotyping primers used in this study. The number of the gene tested in genotyping or qPCR, the name of the gene, the direction of the primer corresponding to the gene, the nucleotide sequence of the primer in 5' → 3' direction and the function of the gene investigated in this study are given.

AGI Code	Gene name	Direction	Sequence (5' - 3') Function	
qPCR primers				
AT4G1351	*AMT1;1*	FW	CGCGGCGCTGACAACCCTAT	Ammonium transporter (AMT)
		RV	GAGGACTAGGGCCGCCACGA	
AT1G64780	*AMT1;2*	FW	GGCCGGTCCGTGGCTTTACG	
		RV	GACCGCGGTGCGACCTACAG	
AT3G24300	*AMT1;3*	FW	CGGCCACTCTGCCTCGCTAG	
		RV	CCGCACACAATCGCTGCCCA	
AT4G26080	*ABI1*	FW	TGGCGGTTCTCAGGTAGCGAACTA	AMT regulation
		RV	GTCTCCGGCGCAACTGACTCAA	
AT4G17615	*CBL1*	FW	CATTGAACGACAAGAGGTCA	
		RV	CTTGATTCACGTCTGCATCT	
AT1G30270	*CIPK23*	FW	CGTTTTGGAATTCGTCACTG	
		RV	TGTTGGAAATACTTCCTCGC	
AT4G27830	*BGLU10*	FW	ACTCTCTACTTTCCGTTTTCTTGG	ABA deglycosylation
		RV	CGCTAGGAGTTCTTCCATCTT	
		FW2	TACCGGTGTTTTCGGAGGAA	
		RV2	ATGGAGTAGCCTCCCACAGA	
AT1G02850	*BGLU11*	FW	AGAGAATGGACAAATGACTCCTC	
		RV	GTCCGATCCTTTCCTGCTGA	
AT1G52400	*BGLU18*	FW	CGATGAAGGATCGTGTCGGT	
		RV	CAGTCGTCCAACTCGGACTC	
AT2G32860	*BGLU33*	FW	ACCAACTCGGCTGGTTTCTT	
		RV	TTGGCGAGAGGGGTAGAGAA	
AT4G05320	*UBQ10*	FW	GGCCTTGTATAATCCCTGATGAATAAG	Ref.-gene
		RV	AAAGAGATAACAGGAACGGAAACATAGT	
AT2G28390	*SAND*	FW	CAGACAAGGCGATGGCGATA	
		RV	GCTTTCTCTCAAGGGTTTCTGGGT	
Genotyping primers				
AT4G26080	*ABI1*	LP	TGAATATAGGAAGTCTGAAGCAAGTG	
		RP	CGAAACAGCATCTTCCATCTC	
AT4G27830	*BGLU10*	LP	TTAACGGTCAAGACAAGACAACGACC	
		RP	ATCCTTTACATGATTTTGCGG	
AT1G02850	*BGLU11*	LP	GTGTTCCCTTGAGGAAGGAAG	
		RP	AAGCAGGCAACAGCTAGAGTG	
AT1G52400	*BGLU18*	LP	TACGGGATGATTGAGAGGATG	
		RP	GCTAGGCAAGACGAGATCATC	
pCSA110		LB1sail	GCCTTTTCAGAAATGGATAAATAGCCTTGC	
pBIN-pROK2		LBb1	GCGTGGACCGCTTGCTGCAACT	
quantitative PCR primers				
AT4G27830	*BGLU10*	FW	TCTTGGCAACAAGCGACAGT	
		RV	TCCTCCGAAAACACCGGTAA	
AT1G02850	*BGLU11*	FW	TTGTCTTTGGCTCTGGCACA	
		RV	TTCTCGATTGATGTGTTTCCAACC	
AT1G52400	*BGLU18*	FW	AAAGCTCCGGGACGTTGTTC	
		RV	CCCTCAGCTTGGAGGTTGGA	
